# Early detection and prognosis evaluation for hepatocellular carcinoma by circulating tumour DNA methylation: A multicentre cohort study

**DOI:** 10.1002/ctm2.1652

**Published:** 2024-05-13

**Authors:** De‐Zhen Guo, Ao Huang, Ying‐Chao Wang, Shuang Zhou, Hui Wang, Xiang‐Lei Xing, Shi‐Yu Zhang, Jian‐Wen Cheng, Ke‐Hui Xie, Qi‐Chang Yang, Cheng‐Cheng Ma, Qing Li, Yan Chen, Zhi‐Xi Su, Jia Fan, Rui Liu, Xiao‐Long Liu, Jian Zhou, Xin‐Rong Yang

**Affiliations:** ^1^ Department of Liver Surgery and Transplantation Liver Cancer Institute Zhongshan Hospital, Fudan University; Key Laboratory of Carcinogenesis and Cancer Invasion (Fudan University), Ministry of Education Shanghai China; ^2^ The United Innovation of Mengchao Hepatobiliary Technology Key Laboratory of Fujian Province Mengchao Hepatobiliary Hospital of Fujian Medical University Fuzhou P. R. China; ^3^ Singlera Genomics Ltd. Shanghai China; ^4^ Biliary Tract Surgery Department IV Eastern Hepatobiliary Surgery Hospital Shanghai China; ^5^ Department of Clinical Pharmacology Xiangya Hospital, Central South University Changsha Hunan China; ^6^ XiangYa Medical Laboratory Central South University Changsha Hunan China

**Keywords:** circulating tumour DNA methylation, diagnosis, hepatocellular carcinoma, liquid biopsy, prognosis

## Abstract

**Background:**

Early diagnosis of hepatocellular carcinoma (HCC) can significantly improve patient survival. We aimed to develop a blood‐based assay to aid in the diagnosis, detection and prognostic evaluation of HCC.

**Methods:**

A three‐phase multicentre study was conducted to screen, optimise and validate HCC‐specific differentially methylated regions (DMRs) using next‐generation sequencing and quantitative methylation‐specific PCR (qMSP).

**Results:**

Genome‐wide methylation profiling was conducted to identify DMRs distinguishing HCC tumours from peritumoural tissues and healthy plasmas. The twenty most effective DMRs were verified and incorporated into a multilocus qMSP assay (HepaAiQ). The HepaAiQ model was trained to separate 293 HCC patients (Barcelona Clinic Liver Cancer (BCLC) stage 0/A, 224) from 266 controls including chronic hepatitis B (CHB) or liver cirrhosis (LC) (CHB/LC, 96), benign hepatic lesions (BHL, 23), and healthy controls (HC, 147). The model achieved an area under the curve (AUC) of 0.944 with a sensitivity of 86.0% in HCC and a specificity of 92.1% in controls. Blind validation of the HepaAiQ model in a cohort of 523 participants resulted in an AUC of 0.940 with a sensitivity of 84.4% in 205 HCC cases (BCLC stage 0/A, 167) and a specificity of 90.3% in 318 controls (CHB/LC, 100; BHL, 102; HC, 116). When evaluated in an independent test set, the HepaAiQ model exhibited a sensitivity of 70.8% in 65 HCC patients at BCLC stage 0/A and a specificity of 89.5% in 124 patients with CHB/LC. Moreover, HepaAiQ model was assessed in paired pre‐ and postoperative plasma samples from 103 HCC patients and correlated with 2‐year patient outcomes. Patients with high postoperative HepaAiQ score showed a higher recurrence risk (Hazard ratio, 3.33, *p* < .001).

**Conclusions:**

HepaAiQ, a noninvasive qMSP assay, was developed to accurately measure HCC‐specific DMRs and shows great potential for the diagnosis, detection and prognosis of HCC, benefiting at‐risk populations.

## Graphical abstract

The HepaAiQ assay integrates the most effective HCC‐specific methylation biomarkers using multilocus quantitative PCR.

In multicentre studies, the HepaAiQ model accurately distinguishes HCC from other liver diseases, surpassing existing serum tests.

The HepaAiQ excels in early‐stage HCC detection in high‐risk patients and post‐resection assessment, potentially fulfilling clinical needs with high accuracy, accessibility and affordability.

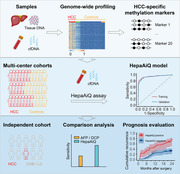

## 1 INTRODUCTION

Liver cancer is the second deadliest cancer in China and the third deadliest worldwide.^1^ Among primary liver cancers, hepatocellular carcinoma (HCC) accounts for 80% of cases.^2^ The incidence and mortality of liver cancer continue to escalate, which constitutes a major global public health burden.^3^ Detecting HCC at curable stages is crucial for effective treatment and improved survival rates.^4^, ^5^, ^6^, ^7^ Unfortunately, current strategies for HCC diagnosis, either serum alpha‐fetoprotein (AFP) or ultrasound, lack sufficient sensitivity and specificity, especially for early‐stage HCC.^8^, ^9^, ^10^ Thus, there is still an urgent need for the development of highly sensitive, cost‐effective diagnostic tool to further improve the prognosis of patients with HCC.

DNA methylation aberrations have become widely recognised as cancer biomarkers.^11^, ^12^, ^13^ Changes in DNA methylation that lead to the dysregulation of gene expression have been identified in many tumours, both early in tumourigenesis and throughout progression.^14^, ^15^ Specifically, the presence of elevated DNA methylation in liver tumours consistently distinguishes patients from healthy controls.^16^, ^17^, ^18^ However, the minute quantities of aberrant DNA released from tumour cells into the blood (circulating tumour DNA, ctDNA) compared to those from numerous healthy cells pose technical challenges for cancer detection, especially at early stages.^19^, ^20^, ^21^, ^22^

Recent technical developments, specifically designed to capture methylation aberrations, have facilitated highly sensitive detection.^23^, ^24^, ^25^ High‐throughput next‐generation sequencing (NGS) techniques have demonstrated the feasibility of using methylation markers for gastrointestinal and hepatobiliary cancers.^26^, ^27^, ^28^ NGS technology is typically integrated with a sophisticated machine‐learning pipeline to detect aberrant patterns of ctDNA in the plasma of patients with HCC.^29^, ^30^, ^31^ However, the widespread adoption of NGS assays in clinical practice is limited by their high operating costs and complexity. Therefore, there is an unmet need for highly accurate, accessible, and pragmatic HCC tests for clinical implementation.

In this study, we sequentially constructed a blood‐based multilocus quantitative methylation‐specific PCR (qMSP) test, HepaAiQ, designed for HCC diagnosis. Employing a stepwise marker elimination approach, we identified highly discriminative methylation markers for HCC and subsequently verified them in independent tumour tissues and plasma samples. HepaAiQ, featuring the best‐performing markers, exhibited accurate and consistent diagnosis of early‐stage HCC in several rounds of blind tests across multiple centres. We further conducted a proof‐of‐concept study to detect ctDNA presence in post‐hepatectomy HCC patients using HepaAiQ and associating it with tumour recurrence. HepaAiQ outperformed existing blood tests, underscoring its potential as an efficient, noninvasive and cost‐effective assay for early diagnosis and prognostic assessment of HCC patients.

## 2 METHODS

### 2.1 Study design

**FIGURE 1:**
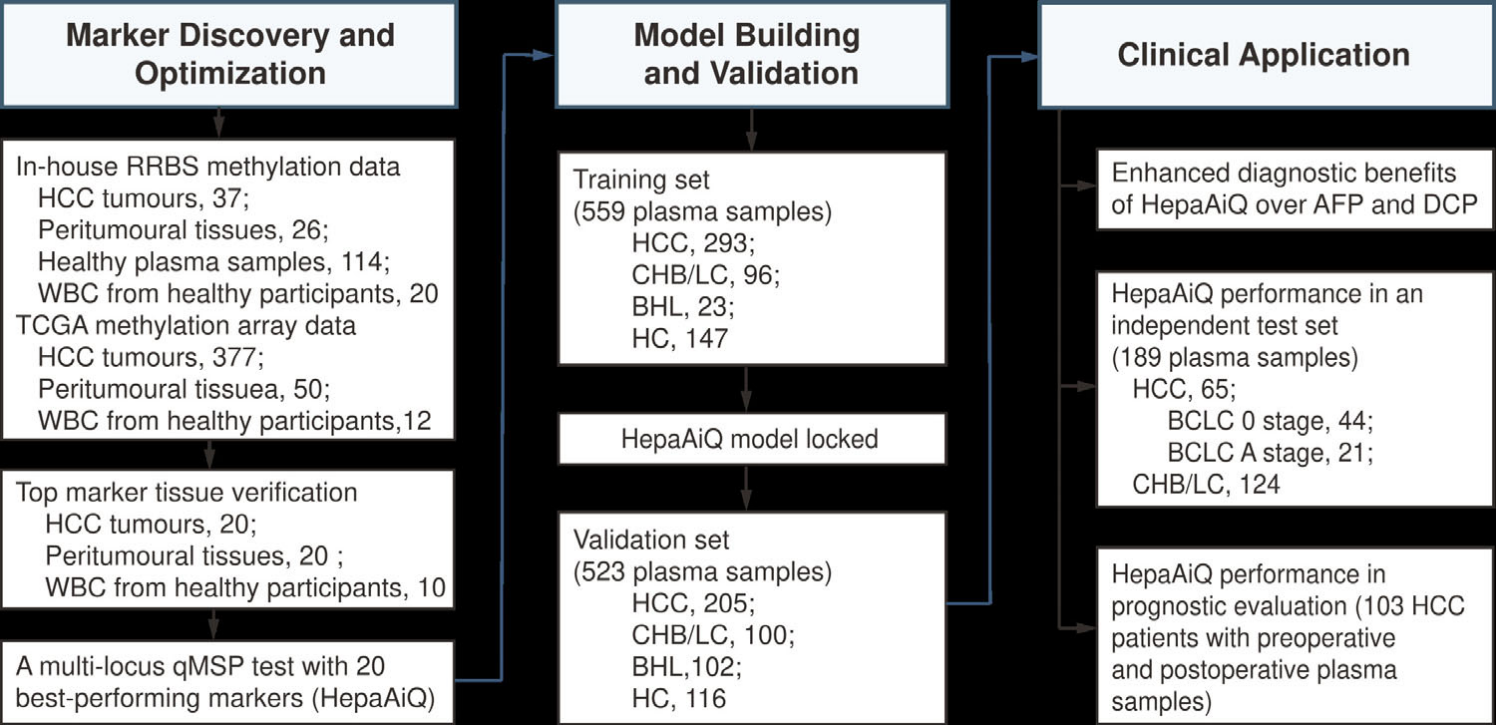
Schematic representation of the study design. The study was designed in three phases: marker discovery and optimisation, model building and validation, and clinical application. First, markers were selected based on publicly available and in‐house genome‐wide methylation datasets, verified using additional tissue samples, and optimised for the 20 best‐performing markers. A blood‐based multilocus qMSP assay, HepaAiQ, was developed using these markers. Second, the HepaAiQ model for early‐stage HCC detection was established in 559 patients and validated in an independent cohort of 523 patients. Finally, the HepaAiQ model was applied for comparison to existing serum assays, HCC detection in an independent test set, and prognostic evaluation in HCC patients. HCC, hepatocellular carcinoma; RRBS, reduced representation bisulphite sequencing; TCGA, The Cancer Genome Atlas; WBC, white blood cell; qMSP, quantitative methylation‐specific PCR; CHB, chronic hepatitis B; LC, liver cirrhosis; BHL, benign hepatic lesion; HC, healthy control; AFP, alpha‐fetoprotein; DCP, des‐gamma‐carboxy prothrombin.

This study was divided into three chronological phases (Figure 1). In the marker discovery phase, in‐house bisulphite sequencing data were generated from 37 HCC tumours, 26 peritumoural tissues, 114 healthy plasma samples and 20 white blood cells (WBCs) from healthy participants. DNA methylation data of 377 HCC tumours and 50 peritumoural tissues publicly accessible through The Cancer Genome Atlas (TCGA) were used to identify specific DNA methylation markers in HCC tumours (TCGA‐LIHC dataset, https://portal.gdc.cancer.gov/). These markers were further verified and ranked based on their ability to distinguish patients with HCC from controls. The 20 best‐performing markers were integrated into a blood‐based multilocus qMSP test, designated HepaAiQ (Table S1). The HepaAiQ model for the diagnosis of HCC was built in 559 patients and blindly validated in 523 patients (Tables 1 and S2). The HepaAiQ model performance was also compared with AFP and des‐gamma‐carboxy prothrombin (DCP), respectively. We further utilised another independent cohort (*n* = 189) to validate the locked HepaAiQ model's ability to distinguish early HCC from the high‐risk group. Finally, the HepaAiQ assay's clinical significance for monitoring treatment response and prognostic evaluation was investigated in an additional cohort of 103 patients with resectable HCC.

**TABLE 1:** Clinical characteristics of HCC in the diagnosis cohort.

Variable	Training (*n* = 559)	Validation (*n* = 523)	*p* Value
**HCC count**	293	205	
Age median (Min, Max)	58.0 (30.0, 81.0)	59.0 (30.0, 81.0)	
Age (years)			.018
≤50	83 (28.3%)	39 (19.0%)	
> 50	210 (71.7%)	166 (81.0%)	
Gender			.386
Male	246 (84.0%)	166 (81.0%)	
Female	47 (16.0%)	39 (19.0%)	
Tumour size (cm)			.761
≤5	179 (61.1%)	128 (62.4%)	
> 5	114 (38.9%)	77 (37.6%)	
Tumour number			.030
Single	248 (84.6%)	187 (91.2%)	
Multiple	45 (15.4%)	18 (8.8%)	
HBsAg			.466
Negative	80 (27.3%)	50 (24.4%)	
Positive	213 (72.7%)	155 (75.6%)	
Child‐Pugh class			.027
A	264 (90.1%)	171 (83.4%)	
B, C	29 (9.9%)	34 (16.6%)	
BCLC stage (CNLC stage)			.180
0–A (I)	224 (76.5%)	167 (81.5%)	
B–D (II–IV)	69 (23.5%)	38 (18.5%)	
AFP (ng/mL)			.074
≤20	119 (40.6%)	101 (49.3%)	
> 20	167 (57.0%)	102 (49.8%)	
No test	7 (2.4%)	2 (1.0%)	
DCP (μg/L)			.822
≤40	34 (11.6%)	29 (14.1%)	
> 40	94 (32.1%)	75 (36.6%)	
No test	165 (56.3%)	101 (49.3%)	
**CHB/LC count**	96	100	
Age median (Min, Max)	56.0 (30.0, 88.0)	53.0 (29.0, 84.0)	
Age (years)			.256
≤50	30 (31.3%)	39 (39.0%)	
> 50	66 (68.8%)	61 (61.0%)	
Gender			.328
Male	67 (69.8%)	76 (76.0%)	
Female	29 (30.2%)	24 (24.0%)	
Child‐Pugh class			.387
A	68 (70.8%)	72 (72.0%)	
B, C	22 (22.9%)	17 (17.0%)	
Unknown	6 (6.3%)	11 (11.0%)	
AFP (ng/mL)			.320
≤20	57 (59.4%)	65 (65.0%)	
> 20	16 (16.7%)	12 (12.0%)	
No test	23 (24.0%)	23 (23.0%)	
DCP (μg/L)			.089
≤40	44 (45.8%)	54 (54.0%)	
> 40	12 (12.5%)	6 (6.0%)	
No test	40 (41.7%)	40 (40.0%)	
**BHL count**	23	102	
Age median (Min, Max)	50.0 (33.0, 71.0)	43.0 (23.0, 74.0)	
Age (years)			.191
≤50	12 (52.2%)	68 (66.7%)	
> 50	11 (47.8%)	34 (33.3%)	
Gender			.125
Male	16 (69.6%)	53 (52.0%)	
Female	7 (30.4%)	49 (48.0%)	
Child‐Pugh class			1.000
A	23 (100%)	102 (100%)	
B, C	0 (0%)	0 (0%)	
AFP (ng/mL)			1.000
≤20	10 (43.5%)	87 (85.3%)	
> 20	0 (0%)	0 (0%)	
No test	13 (56.5%)	15 (14.7%)	
DCP (μg/L)			1.000
≤40	10 (43.5%)	87 (85.3%)	
> 40	0 (0%)	1 (1.0%)	
No test	13 (56.5%)	14 (13.7%)	
**HC count**	147	116	
Age median (Min, Max)	47 (22.0, 83.0)	47.0 (25.0, 81.0)	
Age (years)			.494
≤50	89 (60.5%)	75 (64.7%)	
> 50	58 (39.5%)	41 (35.3%)	
Gender			.442
Male	108 (73.5%)	90 (77.6%)	
Female	39 (26.5%)	26 (22.4%)	

HCC, hepatocellular carcinoma; BCLC, Barcelona Clinic Liver Cancer staging system; CNLC, China Liver Cancer staging system; AFP, alpha‐fetoprotein; DCP, des‐gamma‐carboxy prothrombin; CHB, chronic hepatitis B; LC, liver cirrhosis; BHL, benign hepatic lesion; HC, healthy control.

### 2.2 Patient enrolment and characteristics

The study was registered at https://register.clinicaltrials.gov with the unique identifier NCT05431621. Patients were prospectively enrolled before diagnosis in this multicentre study at three clinical institutions in China from September 2020 to September 2022 (Zhongshan Hospital, Fudan University; Mengchao Hepatobiliary Hospital of Fujian Medical University; and Eastern Hepatobiliary Surgery Hospital). This study obtained approval from the Ethics Committees of the leading centre of Zhongshan Hospital of Fudan University and all participating centres. Written informed consent for the archival of biospecimens and their utilisation in future studies was provided by all patients at the participating institutions.

Patients with HCC were clinically or pathologically diagnosed according to the American Association for the Study of Liver Diseases guidelines.^32^ The diagnosis of chronic hepatitis B (CHB) was confirmed by the presence of hepatitis B surface antigen (HBsAg) for at least 6 months.^33^ The diagnosis of liver cirrhosis (LC) was based on liver biopsy or clinical imaging evidence.^34^ Benign hepatic lesion (BHL) was diagnosed using standard clinical imaging evidence and pathological data. Healthy controls (HCs) were eligible blood donors with normal biochemistry, indicating the absence of liver disease, viral hepatitis or malignancy. Patients with a history of cancer in other organs or those who failed to yield the minimum required volume of plasma were excluded. The Child‐Pugh scoring system was used to assess liver function. Samples were classified and staged following the Barcelona Clinic Liver Cancer (BCLC) stage^35^ and China Liver Cancer (CNLC) Stage Guidelines.^36^

All samples underwent processing at Singlera Genomics following plasma separation. Diagnostic information was included in the training set but omitted from the blinded validation set. The HepaAiQ model training and cross‐validation were conducted using unblinded samples. Subsequently, the model was locked and validated in the blind validation set. In the validation set, all patient information was single blinded to Singlera Genomics, and the HepaAiQ model results were sent back to Zhongshan Hospital, Mengchao Hepatobiliary Hospital of Fujian Medical University, and Eastern Hepatobiliary Surgery Hospital after clinical or pathological diagnosis was obtained. Data analysts would compare the model results with the diagnosis.

An additional cohort of 103 HCC patients was retrospectively enrolled from Mengchao Hepatobiliary Hospital of Fujian Medical University with the following criteria: (1) patients with HCC who underwent curative resection of the tumour^4^; (2) paired plasma samples from each patient were collected before and one month after hepatectomy; (3) patients received routine follow‐up every three months after operation.^4^ In cases where recurrence was suspected, patients underwent abdominal computed tomography (CT), magnetic resonance imaging (MRI) scans, or bone scans. All qualified plasma samples from enrolled patients were examined using the HepaAiQ assay.

### 2.3 Identification of HCC‐specific differentially methylated region (DMR)

The sequencing data from reduced representation bisulphite sequencing (RRBS) libraries generated from tissues and plasma samples were analysed. Publicly available Infinium Human Methylation 450k (HM450) data for tumours and peritumoural tissues were obtained from TCGA. Using these data, three sets of differentially methylated region (DMR) candidates were generated: HCC tumours compared to peritumoural tissue from the RRBS dataset; HCC tumours compared to healthy plasma from the RRBS dataset; and HCC tumours compared to peritumoural tissue from the TCGA dataset. The final DMR candidates were determined as the overlapped set filtered by the median of white blood cells from the RRBS dataset (for detailed methods, see the Supplementary Materials).

### 2.4 Blood sample collection and HepaAiQ assay for plasma ctDNA methylation

Peripheral blood samples (5–10 mL) were individually collected from various centres before the initial diagnosis. Throughout the entire measurement process, diagnostic and clinical information, including the disease status of each sample, was masked until the results were ready for statistical analysis.

Plasma ctDNA was extracted utilising a QIAamp Circulating Nucleic Acid Kit (55114; Qiagen) following the manufacturer's instructions. Samples of 10−20 ng ctDNA were bisulphite converted by EZ DNA Methylation‐Lightning Kit (Zymo Research, D5031). Converted DNA was amplified with a primer pool using the ProFlex™ PCR System (Thermo Fisher Scientific). The preamplified products were analysed by quantitative PCR employing a standard procedure (NovoStart MethyLight qPCR SuperMix, NovoProtein) on an ABI 7500 Real‐Time PCR thermal cycler.

### 2.5 The construction of HepaAiQ model for HCC diagnosis

The incremental feature selection (IFS) method was applied to model building using the Support Vector Regression (SVR) model (Python v3.9.12, scikit‐learn v1.1.2). Markers were individually evaluated using receiver operating characteristic (ROC) curves. First, the marker with the highest area under the ROC curve (AUC) was selected as the anchor marker. Each of the remaining markers was combined with the anchor marker, and a two‐marker combination with the highest mean AUC of fourfold cross‐validation with 10 repetitions was performed. Thus, the third, fourth and fifth markers were selected to determine the best three‐marker, four‐marker, five‐marker, etc. combinations until all 20 markers were included. The best marker combination was determined as that with the highest mean AUC of the cross‐validation among all 20 combinations. The final model was trained using the SVR model with the best marker combination in a training set of 293 HCC patients and 266 controls. The cycle threshold values derived from quantitative PCR of the best marker combination computed a score termed HepaAiQ score that predicted disease status. The cutoff value of the HepaAiQ model was determined to be 0.471 when the AUC was 0.944 and the specificity was 90%. A plasma sample was considered positive when its HepaAiQ score was above the cutoff; otherwise, it was considered negative. The model was then locked and further validated.

### 2.6 Statistical analysis

The model's performance was assessed for sensitivity and specificity. Sensitivity was calculated as true positives divided by the sum of true positives and false negatives, while specificity was calculated as true negatives divided by the sum of true negatives and false positives. The 95% confidence intervals (CI) were calculated using the proportion test. The Mann–Whitney *U* test was employed to identify significant differences between two groups. Comparisons of the two ratios were performed using the chi‐square test and Fisher's exact test. Prognostic statistical analyses were performed using the survival package^37^ in R software v4.1.3. Cumulative recurrence and survival rates were determined utilising the Kaplan–Meier method and assessed using the log‐rank test. Univariate and multivariate analyses were performed using a Cox proportional hazards regression model. All *p* values were considered two‐sided, with values less than .05 deemed statistically significant.

## 3 RESULTS

### 3.1 Study design and patient characteristics

This study was strategically designed in sequential phases to establish and validate the noninvasive HepaAiQ assay to aid in the diagnosis of HCC (see Section 2, Figure 1). The clinical characteristics of patients enrolled in this study were summarised in Table 1. Covariate analysis was performed to examine variables that might impact the HepaAiQ model. To build a model capable of detecting early‐stage cancer, we deliberately enrolled a higher proportion of HCC patients at early stages (BCLC stage 0/A or CNLC stage I). The proportions of patients with stage 0/A were 76.5%, 81.5%, and 100% in the training, validation, and independent test sets, respectively. In addition, the control cohort encompassed patients with various chronic liver diseases, such as chronic viral hepatitis, liver cirrhosis and benign hepatic lesions, ensuring a comprehensive representation of clinical conditions for differential diagnosis.

### 3.2 HCC marker discovery and optimisation

**FIGURE 2:**
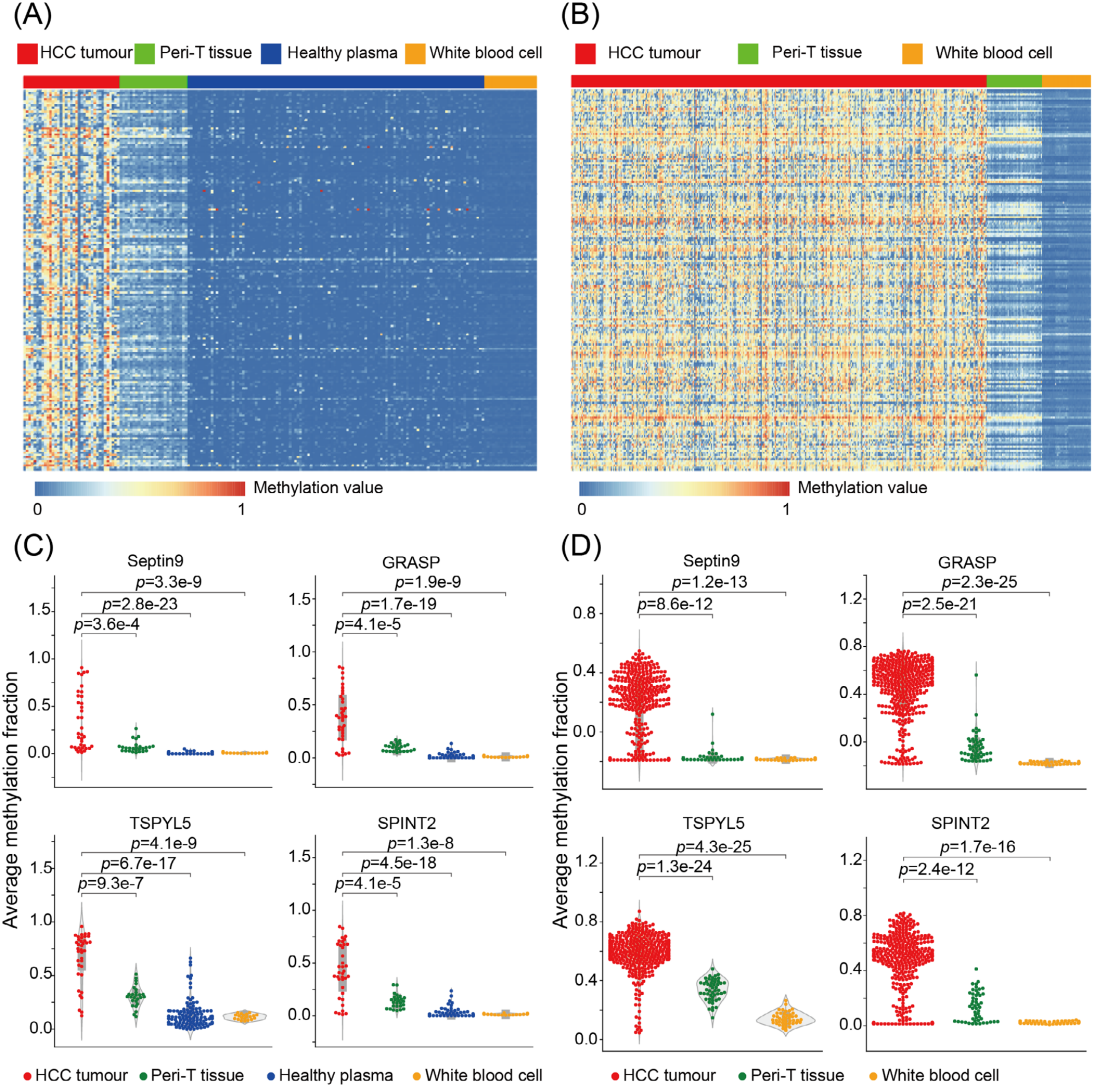
Discovery of hepatocellular carcinoma (HCC) methylation markers. (A) Heatmap displaying in‐house RRBS data comparing 37 HCC tumours to 26 peritumoural tissues, 114 healthy plasma samples, and 20 white blood cell samples from healthy individuals. Each row represented a methylation marker, and each column represented a sample. (B) Heatmap reflecting the TCGA‐LIHC dataset comparing 377 HCC tumours to 50 peritumoural tissues, and 12 white blood cell samples from healthy individuals. Each row represented a methylation marker, and each column represented a sample. (C) The average methylation fraction of four known HCC methylation markers among selected markers in the RRBS dataset was higher in HCC tumours compared to peritumoural tissues, normal plasma and white blood cells. (D) The average methylation fraction of four known HCC methylation markers among selected markers in the TCGA array dataset was higher in HCC tumours compared to peritumoural tissues, normal plasma, and white blood cells. Peri‐T, peritumoural tissues; RRBS, reduced representation bisulphite sequencing; WBC, white blood cell; TCGA, The Cancer Genome Atlas.

To screen for HCC‐specific biomarkers, we conducted genome‐wide methylation profiling of 37 HCC tumours, 26 peritumoural tissues, 114 healthy plasma samples, and 20 white blood cells from healthy individuals using the RRBS approach (see Section 2). The sequencing data yielded 2.8 million CpG sites per sample with an average depth of 27.7× in tissue and 3.6 million CpG sites with 18.2× depth in plasma. Additionally, we obtained array‐based methylation data from the publicly available TCGA database as separate independent sources of markers. Through three independent comparisons of HCC tumours against peritumoural tissue or healthy plasma (Figure S1), we ranked DMRs highly represented in HCC tumours based on the adopted *p* value, methylation quantiles, and CG enrichment to filter out markers with higher background noise. Finally, we identified 183 common hypermethylated DMRs in cancer samples compared to all other controls, irrespective of the methodology (Figure 2A and B, see Supplementary Methods in Supplementary Material). Several of these markers have been previously reported to be relevant in tumourigenesis^38^, ^39^, ^40^ (Figure 2C and D), confirming the efficacy of the marker selection. After curation of the genomic location, gene annotation and literature‐based evidence, we assessed the analytical accuracy of the candidate markers by qMSP in an additional 20 HCC tumours, 20 peritumoural tissues, and 10 WBCs from healthy participants. To eliminate DMRs with insufficient signal amplification, lower distinguishing power, or lower fold changes, we selected the 20 most effective markers and incorporated them into a multilocus qMSP assay, designated as HepaAiQ (Table S1).

### 3.3 Formulating and validating HepaAiQ model

**FIGURE 3:**
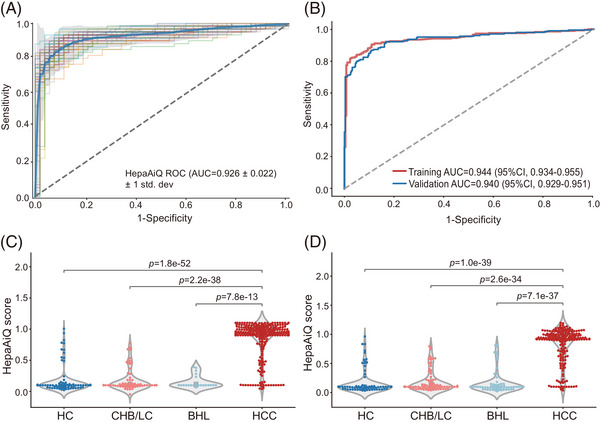
HepaAiQ model training and validation in plasma. (A) The ROC curve of the HepaAiQ model across 40 iterations of cross‐validation in the training set. (B) The ROC curve of the HepaAiQ model in the training and validation sets. (C) The HepaAiQ scores were significantly higher in patients with 293 HCC than in the 266 control groups in the training set. (D) The HepaAiQ scores were significantly higher in patients with 205 HCC than in the 318 control groups in the validation set. ROC, receiver operating characteristic; AUC, area under curve; HC, healthy control; CHB, chronic hepatitis B; LC, liver cirrhosis; BHL, benign hepatic lesion; HCC, hepatocellular carcinoma; CI, confidence interval.

To build the HepaAiQ model for HCC diagnosis, we enrolled a training cohort comprising 293 HCC patients, 96 patients with CHB/LC, and 23 BHL patients, along with 147 HCs from multiple centres (see Section 2, Figure 1, Table 1). To enhance the diagnostic model for early‐stage HCC, we intentionally increased the proportion of HCC patients at BCLC stage 0/A (CNLC stage I) to 76.5%. All 559 plasma samples were processed using the HepaAiQ assay. A model‐building approach with incremental feature selection was used to assess all possible marker combinations and determine the optimal model performance (see Section 2). Cross‐validation of HepaAiQ performance over 40 iterations of the training set led to an average AUC of 0.926 ± 0.022 (Figure 3A). The resultant HepaAiQ classifier achieved an AUC of 0.944 (95% CI: 0.934–0.955) (Figure 3B), corresponding to a sensitivity of 86.0% (95% CI: 82.0%−90.0%) in HCC and a specificity of 90.6% (95% CI: 84.8%−96.5%), 100% (95% CI: 85.7%−100.0%) and 91.8% (95% CI: 87.4%−96.3%) in CHB/LC, BHL and HC, respectively (Table 2).

**TABLE 2:** Performance metrics of the HepaAiQ model in plasma samples.

	Total	Negative	Positive	Sensitivity (95% CI)	Specificity (95% CI)	PPV (95% CI)	NPV (95% CI)
**Plasma (training set)**						92.3% (89.1%−95.5%)	85.7% (81.6%−89.7%)
All HCCs	293	41	252	86.0% (82.0%−90.0%)	**–**		
BCLC 0‐A (CNLC I)	224	38	186	83.0% (78.1%−88.0%)	–		
BCLC B–D (CNLC II–IV)	69	3	66	95.7% (88.0%−98.5%)	–		
All controls	266	245	21	**–**	92.1% (88.9%−95.3%)		
CHB/LC	96	87	9	–	90.6% (84.8%−96.5%)		
BHL	23	23	0	–	100.0% (85.7%−100.0%)		
HC	147	135	12	–	91.8% (87.4%−96.3%)		
**Plasma (validation set)**						84.8% (79.9%−89.7%)	90.0% (86.7%−93.3%)
All HCCs	205	32	173	84.4% (79.4%−89.4%)	**–**		
BCLC 0‐A (CNLC I)	167	32	135	80.8% (74.9%−86.8%)	–		
BCLC B–D (CNLC II–IV)	38	0	38	100.0% (90.8%−100.0%)	–		
All controls	318	287	31	**–**	90.3% (87%−93.5%)		
CHB/LC	100	88	12	–	88.0% (81.6%−94.4%)		
BHL	102	94	8	–	92.2% (86.9%−97.4%)		
HC	116	105	11	–	90.5% (85.2%−95.8%)		
**Plasma (independent test set)**						78.0% (67.4%−88.5%)	85.4% (79.3%−91.5%)
All HCCs	65	19	46	70.8% (59.7%−81.8%)			
BCLC 0‐A (CNLC I)	65	19	46	70.8% (59.7%−81.8%)			
All controls	124	111	13	–	89.5% (84.1%−94.9%)		
CHB/LC	124	111	13	–	89.5% (84.1%−94.9%)		

HCC, hepatocellular carcinoma; BCLC, Barcelona Clinic Liver Cancer staging system; CNLC, China Liver Cancer staging system; AFP, alpha‐fetoprotein; DCP, des‐gamma‐carboxy prothrombin; CHB, chronic hepatitis B; LC, liver cirrhosis; BHL, benign hepatic lesion; HC, healthy control; CI, confidence interval.

To evaluate the model's robustness and stability, we assembled a validation cohort of 523 patients (HCC, 205; CHB/LC, 100; BHL, 102; HC, 116) and processed their samples with HepaAiQ in a single‐blind manner (see Section 2, Table 1). In this cohort, 81.5% of HCC patients presented at early stages (BCLC stage 0/A or CNLC stage I). This cohort also expanded the representation of patients with benign hepatic lesions (such as haemangiomas, hepatic cysts, and focal nodular hyperplasia) commonly encountered in clinical settings, necessitating differential diagnosis. As expected, HepaAiQ performed consistently in the validation set, achieving an AUC of 0.940 (95% CI: 0.929–0.951) (Figure 3B), with sensitivity of 84.4% (95% CI: 79.4%−89.4%) and specificities of 88.0% (95% CI: 81.6%−94.4%), 92.2% (95% CI: 86.9%−97.4%), and 90.5% (95% CI: 85.2%−95.8%) for CHB/LC, BHL and HC, respectively (Table 2).

Notably, the HepaAiQ model achieved sensitivities of 83.0%/80.8% for early‐stage HCC, 75.6%/77.8% for tumours less than 2 cm, and 83.9%/82.9% for single tumour in the training/validation sets, respectively (Figures S2 and S3). Patients with HCC exhibited significantly higher HepaAiQ scores than controls (*p *< .001) (Figure 3C and D). Additionally, we conducted a covariance analysis of patient characteristics to identify factors that could potentially affect HepaAiQ performance. No significant correlations were observed between the HepaAiQ score and age, sex or Child‐Pugh grading in either the training set (Figures S4 and S5) or validation sets (Figures S6 and S7). All of these findings demonstrate a strong potential of the HepaAiQ model for HCC early detection and differential diagnosis.

### 3.4 Comparison of HepaAiQ model with the traditional biomarkers AFP and DCP

**FIGURE 4:**
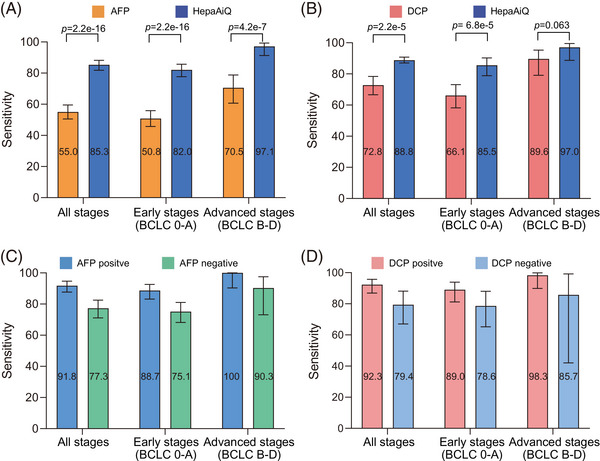
Performance comparison of HepaAiQ, AFP and DCP in detecting HCC. (A) The sensitivity of HepaAiQ model in 489 HCC patients is significantly higher than that of AFP across the cancer stage. (B) The sensitivity of HepaAiQ model in 232 HCC patients is significantly higher than that of DCP across the cancer stage. (C) The sensitivity of HepaAiQ model is comparable between 269 AFP‐positive and 220 AFP‐negative HCC patients across the cancer stage. (D) The sensitivity of HepaAiQ model is comparable between 169 DCP‐positive and 63 DCP‐negative HCC patients across the cancer stage. HCC, hepatocellular carcinoma; AFP, alpha‐fetoprotein; DCP, des‐gamma‐carboxy prothrombin; AUC, area under the curve.

We further investigated whether the HepaAiQ model could offer additional clinical advantages over existing blood tests for HCC diagnosis. In 489 HCC patients examined with both HepaAiQ and AFP tests, HepaAiQ significantly outperformed the AFP test (85.3% vs. 55%), while comparable specificities were shown in 247 controls (90.3% vs. 88.7%) (Table S2, Figure 4A). Importantly, HepaAiQ achieved a sensitivity of 75.1% in 189 HCC patients at early stages who tested negative for AFP (Table S3, Figure 4C). The HepaAiQ score was independent of the status of the well‐known HCC marker, AFP, although patients with AFP‐positive HCC tended to have a higher HepaAiQ score.

Similarly, a subgroup of 441 patients was examined using the DCP, another HCC blood test. A superior performance of HepaAiQ over DCP was observed in 232 HCC samples and 209 controls (Figure 4B, Table S4). Noteworthy, HepaAiQ detected 44 (78.6%) of 56 early‐stage HCC patients who tested negative with DCP (Figure 4D, Table S5). These observations strongly suggest that methylation markers can accurately capture HCC signals compared to traditional protein markers, potentially improving the effectiveness of surveillance care for HCC in clinical settings.

### 3.5 Independent test of HepaAiQ model to distinguish early HCC in high‐risk populations

Ultrasonography combined with AFP is frequently used in detecting HCC within high‐risk populations. To simulate the real‐world performance of the HepaAiQ model, we recruited an independent test cohort of 189 patients with chronic liver diseases, either with HCC (BCLC stage 0/A or CNLC stage I, 65) or without HCC (CHB/LC, 124) (Table S6). HepaAiQ model resulted in a sensitivity of 70.8% (95% CI: 59.7%−81.8%) in early‐stage HCC and a specificity of 89.5% (95% CI: 84.1%−94.9%) in CHB/LC (Table 2), compared to a sensitivity of 58.5% (95% CI: 46.5%−70.4%) and a specificity of 92.7% (95% CI: 87.9%−97.6%) by AFP in the same set (Table S7). Although with a limited size cohort, HepaAiQ was suggested to be a promising model for HCC surveillance in high‐risk populations.

### 3.6 The prognostic significance of HepaAiQ model in HCC patients undergoing resection

**FIGURE 5:**
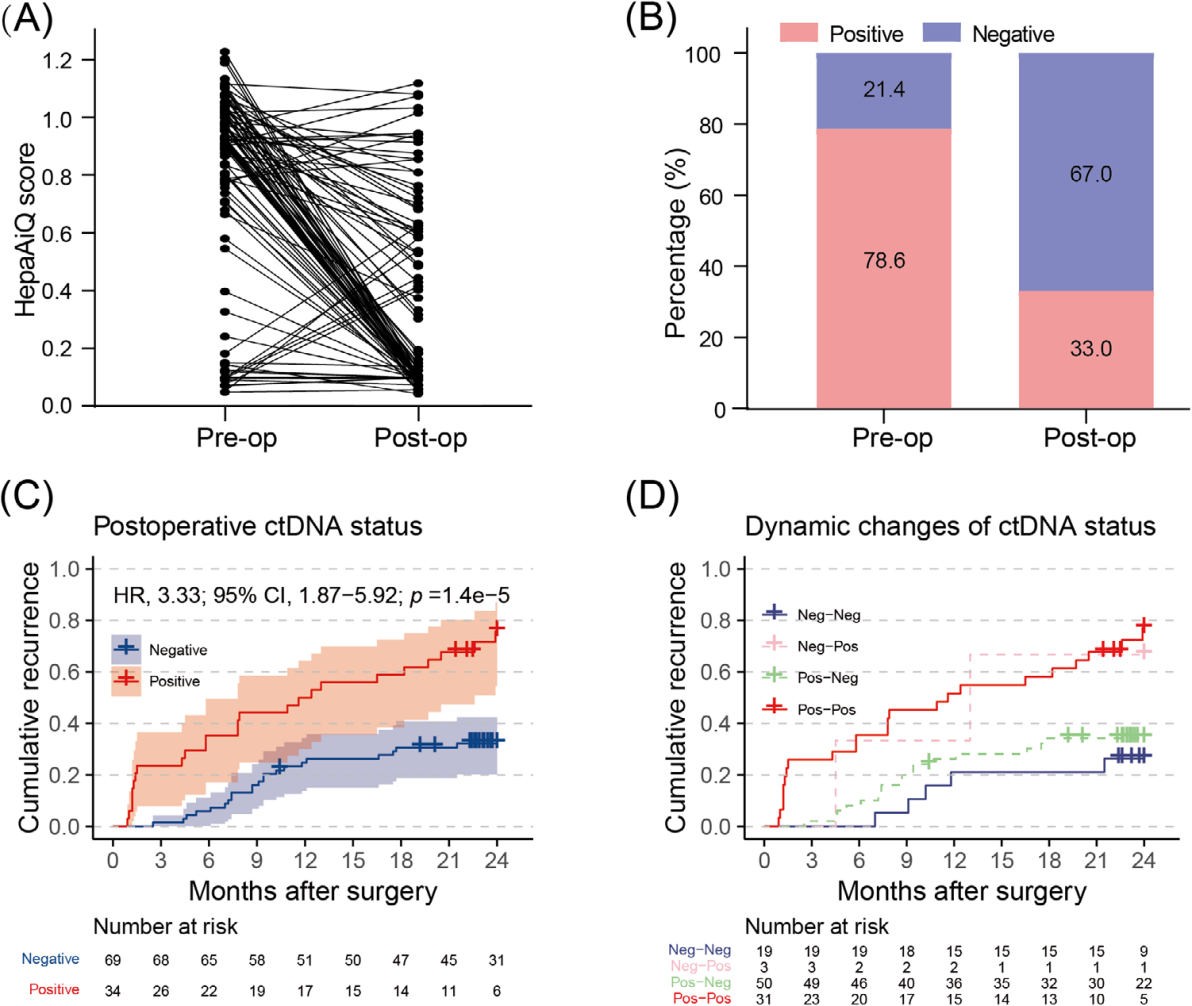
Prognostic assessment of HepaAiQ model. (A) HepaAiQ scores of HCC patients before and after surgery. (B) Percentage of HepaAiQ positivity in HCC patients before and after surgery. (C) Kaplan–Meier curves displaying cumulative events of recurrence based on postoperative ctDNA status. (D) Kaplan–Meier curves illustrating perioperative dynamic changes of ctDNA status. Pre‐op, preoperation; Post‐op, postoperation; Neg‐Neg, Negative to negative; Neg‐Pos, Negative to positive; Pos‐Neg, Positive to Negative; Pos‐Pos, positive to positive; HR, Hazard ratio; CI, confidence interval.

Next, the potential utility of the HepaAiQ model in the prognostic evaluation was investigated in 103 HCC patients who had undergone resection (see Section 2, Table S8). Among these patients, 47 were confirmed to have tumour relapse during postoperative follow‐up, while 56 showed no sign of relapse. Paired perioperative plasma samples had been collected before (mean, 1 day) and after (mean, 34.9 days) surgical resection. We found that the level of ctDNA methylation tested by the HepaAiQ assay significantly decreased in HCC patients one month after tumour resection (Figure 5, Figure S8), with the positive rate decreasing from 78.6% (81/103) to 33.0% (34/103) (Figure 5B). Further analysis revealed that patients with postoperative recurrence exhibited higher positivity in the postoperative HepaAiQ results than did the recurrence‐free ones (Figure S9; *p *< .001). Kaplan–Meier survival analysis indicated that patients with positive postoperative ctDNA had unfavourable outcomes compared with those with negative status (HR, 3.33; 95% CI: 1.87–5.92, *p* < .001), especially for the patients with continuous positive results (Figure 5C and D). Univariate and multivariate analyses confirmed that the postoperative HepaAiQ status was an independent indicator of postoperative recurrence (Table S9).

## 4 DISCUSSION

Owing to the lack of specific symptoms, most patients with HCC reach intermediate and advanced stages at the time of diagnosis and may not be eligible for curative surgical resection.^35^, ^36^ Thus, effective early screening and diagnosis play a pivotal role in improving disease prognosis. Currently, AFP and ultrasound are the primary tools for the detection of HCC, despite their limited diagnostic sensitivity and specificity.^7^, ^8^, ^9^, ^10^ In the present study, we constructed a pragmatic qMSP assay, HepaAiQ, incorporating the most effective methylation biomarkers specific to HCC. HepaAiQ accurately distinguished patients with HCC from individuals with CHB/LC, indicating its ability to differentiate HCC from populations at risk. More importantly, HepaAiQ showed favourable diagnostic sensitivity in HCC patients at BCLC stage 0/A (CNLC stage I). In comparison to routinely used biomarkers such as AFP and DCP, HepaAiQ presented superior diagnostic accuracy, especially in discriminating early‐stage HCC from high‐risk individuals. HepaAiQ also holds promise as a surveillance tool for evaluating the treatment response and prognosis in patients with HCC.

In recent years, HCC‐specific aberrant methylation has been revealed due to the development of NGS technology, which lays the foundation for investigating the potential of ctDNA methylation biomarkers for the detection of HCC.^19^, ^41^, ^42^, ^43^ However, most of these models use NGS assays with high economic and time costs, which hampers their clinical application and population‐level screening. In this study, the HepaAiQ model was built based on the qMSP assay and achieved diagnostic performance comparable to that of previously reported models using NGS. This assay has great advantages in testing cost and detection efficiency, thus facilitating clinical practice at the population level.

We intended to assess the potential benefits of HepaAiQ applied as a surveillance approach in high‐risk population for liver cancer. A Monte–Carlo simulation was conducted to compare the performances of HepaAiQ with the recommended surveillance strategy (ultrasonography combined with AFP test) (for detailed methods, see the Supplementary Material). With reported surveillance compliance, test performance, and prevalence of cirrhosis and viral hepatitis in China,^10^, ^44^, ^45^, ^46^, ^47^, ^48^ Monte–Carlo simulation revealed that HepaAiQ could lead to a 2.5‐fold (95% CI: 1.8–3.4) increase in HCC detection compared to ultrasonography combined with AFP (Figure S10). Moreover, PPV would be expected to increase from 15.2% (95% CI: 9.0%−24.3%) with ultrasonography‐AFP to 26.1% (95% CI: 15.8%−41.8%) with HepaAiQ, whereas the false negative rate decreased from 37.0% (95% CI: 28.1%−46.3%) to 17.8% (95% CI: 13.9%−22.1%), respectively. Therefore, simulation analyses further support HepaAiQ could bring potential benefits to the population at risk of HCC.

Furthermore, most patients showed significantly decreased ctDNA methylation levels after hepatectomy, suggesting that HepaAiQ can sensitively detect changes in tumour load during the course of the disease. Patients who relapsed after surgery had significantly higher postoperative ctDNA methylation levels than those without tumour recurrence, implying the possibility of residual lesions in HCC patients. The postoperative (1‐month after surgery) HepaAiQ score was able to predict tumour recurrence after surgery, which is difficult to predict in clinical settings. These findings provide a glimpse into the prognostic evaluation of HepaAiQ and highlight its potential as an alternative indicator for monitoring treatment response during HCC management.

The HepaAiQ presents several advantages over existing methylation diagnostic assays. First, its utilisation of the qMSP assay significantly reduces both economic and time costs while maintaining comparable diagnostic performance to NGS‐based assays. This positions HepaAiQ as an ideal tool for HCC diagnosis in clinical practice and HCC screening at the community level. Second, the HepaAiQ model demonstrates favourable performance in diagnosing early‐stage HCC and distinguishing patients with HCC from high‐risk populations, suggesting its efficacy as a biomarker in the surveillance of high‐risk populations. In addition, the HepaAiQ model not only aids in HCC detection but also recurrence risk stratification for postoperative patients. As far as we know, HepaAiQ is the first qMSP‐based methylation model to encompass various clinical utilities, including detection, differential diagnosis and prognostic evaluation of HCC.

There are some limitations in this study. All the patients included in this study were of Chinese origin, with the predominant etiology being CHB/LC. Sensitivities are comparable in HCC cases caused by viral hepatitis or other etiologies (alcoholic and nonalcoholic steatohepatitis) across training, validation and test sets (Figure S11). Further studies with diverse populations and other risk factors are required to validate these findings. Additionally, the prognostic value of HepaAiQ was investigated in a cohort with a limited sample size and a short follow‐up period. Therefore, prospective studies with larger sample sizes and long‐term surveillance are required for further validation.

## 5 CONCLUSION

In conclusion, we have developed and validated a novel blood‐based qMSP assay targeting ctDNA methylation, designated HepaAiQ, for differential diagnosis and prognostic assessment of HCC patients. HepaAiQ has the potential to be a cost‐effective and simplified tool for HCC screening at the population level and for improving patient management.

## AUTHOR CONTRIBUTIONS

DZG, AH, YCW and SZ are joint first authors. JZ, XRY, XLL and RL obtained funding. JZ, XRY, XLL and RL designed the study. DZG, AH, YCW, XLX and SYZ collected the data. DZG, AH, YCW, QL, JWC and YC were involved in data cleaning, follow‐up and verification. SZ, HW, KHX, QCY, CCM, ZXS and RL analysed the data. DZG, SZ and RL drafted the manuscript. JZ, XRY, JF, RL and XLL contributed to the interpretation of the results and critical revision of the manuscript for important intellectual content and approved the final version of the manuscript. All authors have read and approved the final manuscript. JZ, XRY, XLL and RL are the study guarantors

## CONFLICT OF INTEREST STATEMENT

Rui Liu reports stock ownership in Singlera Genomics and is an employee of Singlera Genomics. Shuang Zhou, Hui Wang, Ke‐Hui Xie, Qi‐Chang Yang, Cheng‐Cheng Ma and Zhi‐Xi Su are employees of Singlera Genomics. All other authors declare no competing interests.

## FUNDING INFORMATION

This project was supported by grants from The National Key Research and Development Program of China (2019YFC1315800, 2019YFC1315802 and 2021YFC2501900), the State Key Program of National Natural Science of China (81830102), the Original Discovery Program of National Natural Science of China (82150004), the National Natural Science Foundation of China (82072715 and 82341027), the Shanghai Municipal Health Commission Collaborative Innovation Cluster Project (2019CXJQ02), the Eastern Talent Program (Leading project), the Shanghai Municipal Health Commission (201940075 and 2022LJ005), the Shanghai Science and Technology Commission (21140900300 and 22S31901800), the project from Shanghai Hospital Development Center (SHDC2023CRD025), the Projects from Science Foundation of Zhongshan Hospital, Fudan University (2021ZSCX28, 2020ZSLC31).

## ETHICS STATEMENT

This study was approved by the Ethics Committees of the leading centre of Zhongshan Hospital of Fudan University and of all participating centres (No. B2020‐299).

## Supporting information

Supporting Information

## Data Availability

The processed methylation data that support the findings of this study are openly available in OMIX, China National Center for Bioinformation (Beijing, China) at https://ngdc.cncb.ac.cn/omix/releaseList, BioProject ID PRJCA016185.
