# Supplementary material for: Early detection and prognosis evaluation for hepatocellular carcinoma by circulating tumour DNA methylation: A multicentre cohort study
Source: Clin Transl Med. 2024 May 13;14(5):e1652. doi: 10.1002/ctm2.1652 (PMC11091019; doi:10.1002/ctm2.1652)
Supplement: Supplementary file 1 — Supporting Information [file CTM2-14-e1652-s001.docx]

**Supplementary appendix**

**Supplement to: Early detection and prognosis evaluation for hepatocellular carcinoma by circulating tumor DNA methylation: a multi-center cohort study**

**Supplementary methods**

Supplemental method 1. Average methylation fraction (AMF) calculation

Supplemental method 2. OE ratio calculation

Supplemental method 3. Definition of windows in reduced representation bisulfite sequencing (RRBS) dataset

Supplemental method 4. Marker selection in hepatocellular carcinoma (HCC) tumor and peritumoral tissues in RRBS dataset

Supplemental method 5. Marker selection in HCC and healthy plasma in the RRBS dataset

Supplemental method 6. Definition of windows in Candidate windows in The Cancer Genome Atlas (TCGA) dataset

Supplemental method 7. Marker selection in the TCGA dataset

Supplemental method 8. Intersection of markers selected from RRBS and TCGA

Supplemental method 9. Monte Carlo simulation

**Supplementary figures**

Figure S1. Workflow of candidate markers selected from RRBS (reduced representation bisulfite sequencing) and TCGA (The Cancer Genome Atlas) array datasets.

Figure S2. Correlation analysis and sensitivity comparison for tumor stage, tumor size and tumor number of hepatocellular carcinoma patients in the training cohort.

Figure S3. Correlation analysis and sensitivity comparison for tumor stage, tumor size and tumor number of hepatocellular carcinoma patients in the validation cohort.

Figure S4. Correlation analysis of HepaAiQ score with age and gender in the training cohort.

Figure S5. Correlation analysis and sensitivity comparison for Child-Pugh grading of hepatocellular carcinoma patients in the training cohort.

Figure S6. Correlation analysis of HepaAiQ score with age and gender in the validation cohort.

Figure S7. Correlation analysis and sensitivity comparison for Child-Pugh grading of hepatocellular carcinoma patients in the validation cohort.

Figure S8. HepaAiQ scores change in each recurrent patient and non-recurrent patient.

Figure S9. Overall HepaAiQ score changes before and after surgery between patients with and without recurrence.

Figure S10. Modeling the implementation of HepaAiQ in the HCC test.

Figure S11. Sensitivity of HepaAiQ model in HCC cases from different etiologies.

**Supplementary tables**

Table S1. Top 20 best-performing markers selected from quantitative methylation-specific PCR.

Table S2. The performance of AFP and HepaAiQ.

Table S3. The performance of HepaAiQ in AFP-Positive and AFP-negative samples.

Table S4. The performance of DCP and HepaAiQ.

Table S5. The performance of HepaAiQ in DCP-Positive and DCP-negative samples.

Table S6. Patient demographics and clinical information in the independent test cohort.

Table S7. The performance of AFP and HepaAiQ in the independent test cohort.

Table S8. Clinical characteristics of prognosis assessment cohort.

Table S9. Univariate and multivariate Cox regression analysis for possible variables associated with recurrence in prognosis assessment cohort.

**Supplementary method**

**Average methylation fraction (AMF) calculation**

AMF is the measurement of the methylation level of regions.

AMF is defined as the fraction of methylated CpGs of total CpGs in a region, which is calculated as:

$${AMF}_{i}=\frac{M_{i}}{N_{i}}$$

Where i is the target region, N_i_ is the number of CpGs detected in the region. M_i_ is the number of methylated CpGs detected in the region.

**OE ratio calculation**

OE ratio is defined as the observed CpG rate divided by the expected CpG rate.

$${OE}_{i}=\frac{{CpG}_{i}}{\frac{C_{i}\times G_{i}}{L_{i}}}$$

Where i is the target region, CpGi is the number of CpGs in the region. C_i_ is the number of C in the region. G_i_ is the number of G in the region. L_i_ is the length of the region.

**Definition of windows in reduced representation bisulfite sequencing (RRBS) dataset**

Windows in RRBS data were defined as genomic regions covered by most of the samples (37 HCC tumors, 26 peritumoral tissues, 114 normal plasma, and 20 white blood cell samples from healthy individuals) (**Figure S1**). The processed methylation data of RRBS was openly available in [OMIX, China National Center for Bioinformation (Beijing, China)] at https://ngdc.cncb.ac.cn/omix/releaseList, BioProject ID [PRJCA016185]. CpGs covered ≥10x were extracted from each sample. The CpGs appeared in more than 60% of the samples were selected as seed CpGs. A region of 200 base pairs downstream of each seed CpGs was taken as candidate windows, resulting in 2428093 candidate windows when comparing HCC tumors with peritumoral tissues and 1366250 candidate windows when comparing HCC tumors with healthy plasma samples in the RRBS data. AMF of these windows for each sample was calculated as their measurements.

**Marker selection in hepatocellular carcinoma (HCC) tumor and peritumoral tissues in RRBS dataset**

When comparing HCC tumors with peritumoral tissues in the RRBS dataset, the following steps were taken: 1) The Mann-Whitney U test was conducted to compare the AMF between 37 HCC tumors and 26 peritumoral tissues. 308205 windows exhibiting higher AMF in HCC tumor samples compared to peritumoral tissues, with the smallest 40% p-values, were selected for downstream marker selection. 2). The 90th percentiles of AMF were calculated for each window in both HCC tumors and peritumoral tissues respectively. We combined the 90th percentile of AMF from 308205 windows in peritumoral tissues and selected the 80th percentile from these values as the normal cutoff. Similarly, we combined the 90th percentile of AMF from 308205 windows in HCC tumors and selected the 30th percentile from these values as the cancer cutoff. Windows were selected for further analysis if the 90th percentile of AMF in peritumoral tissue samples was smaller than the normal cutoff, and the 90th percentile of AMF in HCC tumor samples exceeded the cancer cutoff. 3). OE ratio and C/G count were calculated. Windows with the highest 90% OE ratio and C/G count greater than 120 but less than 170 were selected as HCC markers.

**Marker selection in HCC and healthy plasma in the RRBS dataset**

When comparing HCC tumors with healthy plasma samples in the RRBS dataset, the following steps were taken: 1) The Mann-Whitney U test was conducted to compare the AMF between 37 HCC tumors and 114 healthy plasma samples. 323294 windows exhibiting higher AMF in HCC tumors compared to healthy plasma samples, with the smallest 50% p-values, were selected for downstream marker selection. 2). The 90th percentiles of AMF were calculated for each window in both HCC tumor and healthy plasma samples respectively. We combined the 90th percentile of AMF from 323294 windows in peritumoral tissues and selected the 90th percentile from these values as the normal cutoff. Similarly, we combined the 90th percentile of AMF from 323294 windows in HCC tumors and selected the 60th percentile from these values as the cancer cutoff. Windows were selected for further analysis if the 90th percentiles of AMF in healthy plasma samples were smaller than the normal cutoff, and the 90th percentile of AMF in HCC tumor samples exceeded the cancer cutoff. 3). OE ratio and C/G count were calculated. Windows with the highest 90% OE ratio and C/G count greater than 120 but less than 170 were selected as HCC markers.

**Definition of windows in Candidate windows in The Cancer Genome Atlas (TCGA) dataset**

When comparing HCC tumors with peritumoral tissues in the TCGA dataset, DNA methylation data for 377 HCC tumors and 50 peritumoral tissues were downloaded from TCGA using the Infinium Methylation 450K assay (**Figure S1**). Data for white blood cells (WBCs) were downloaded from GSE35069 (n=12, 450K), GSE40005 (n=12, 450K), and GSE68777 (n=20, 450K), respectively. We segmented the genome into 313,797 windows with a length of 200bp covered ≥1 CpG in 450K.

**Marker selection in the TCGA dataset**

The methylation level of each window was calculated as the average value of the total CpGs methylation within that window. Windows were excluded if the median methylation level was >0.2 in 12 WBC samples, >0.4 in 50 peritumoral tissues, or <0.28 in 377 HCC tumors. We retained those windows if OE >0.5, the number of CpG >10, and the number of C and G >120. A Wilcoxon rank sum test was conducted to compare the methylation level for each window between HCC tumor and peritumoral tissue samples, as well as between HCC tumor and WBC samples. The selected windows with a difference in median methylation level >0.1, were ranked based on the P value between HCC tumor and peritumoral samples. The top 1200 windows were selected as the final markers.

**Intersection of markers selected from RRBS and TCGA**

Intersections of windows from the RRBS dataset and TCGA dataset were selected. The median of AMF in WBC samples from RRBS data was calculated. The final candidates were determined as the windows with a median of the AMF in WBC samples not exceeding 0.02.

**Monte Carlo simulation**

Monte Carlo simulations were used to compare the HepaAiQ approach to ultrasonography combined with AFP in a theoretical population[1]. Ultrasonography combined with AFP was reported to detect early-stage HCC with 63% sensitivity (95% CI: 48%-75%) and 84% specificity (95% CI: 77% – 89%)[2]. We applied prior probability distributions to simulate the prevalence of cirrhosis, viral hepatitis, and the co-occurrence of these diseases with HCC regarding regional differences in China[3, 4]. We modeled multinomial probabilities for the prevalence of viral hepatitis, cirrhosis, viral hepatitis + HCC, cirrhosis + HCC, and viral hepatitis + cirrhosis + HCC from a Dirichlet with parameters 890, 68, 18, 6, and 18, respectively. Reported or estimated parameters including prevalence rate, adherence rate, sensitivity, and specificity for both HepaAiQ and ultrasonography combined with AFP were used to establish prior predictive probability distributions (beta distributions) in the R package epiR[2, 5-7]. Monte Carlo simulation was repeated 1000 times to calculate PPV= (true positives)/(true positives + false positives), and false negative rate = false negatives / (false negatives + true positives).

**Supplementary figures**

**Figure S1. Workflow of candidate markers selected from RRBS (reduced representation bisulfite sequencing) and TCGA (The Cancer Genome Atlas) array datasets.** HCC, hepatocellular carcinoma; Peri-T, peritumoral tissue; WBC, white blood cell; OE, the observed CpG rate divided by the expected CpG rate.


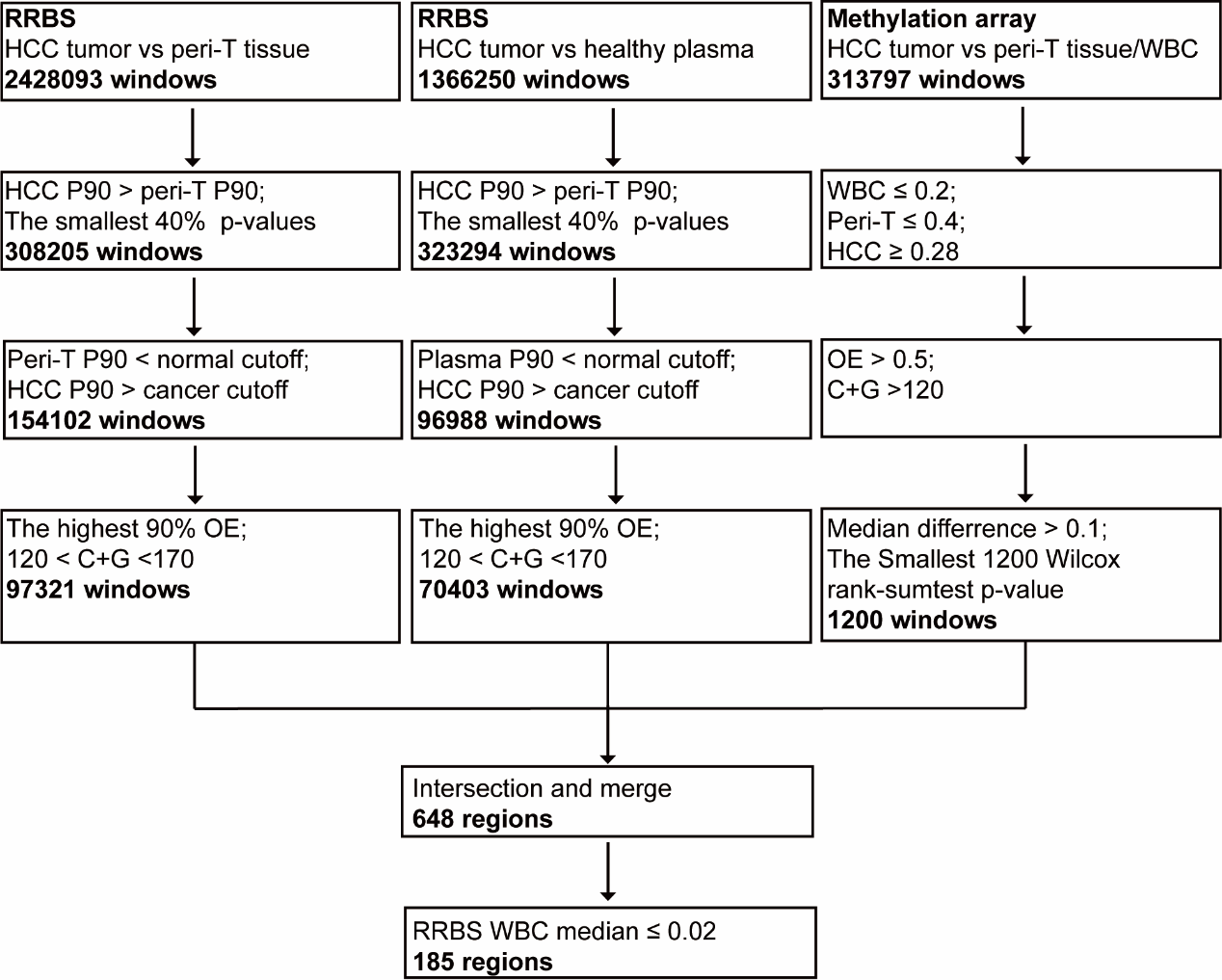


**Figure S2. Correlation analysis and sensitivity comparison for tumor stage, tumor size and tumor number of hepatocellular carcinoma patients in the training cohort.** HepaAiQ score and sensitivity are higher in HCC patients with advanced stage (A, B). No significant correlations were observed between the HepaAiQ score and tumor size (C). Sensitivity is higher in HCC patients with tumor above 5cm (D). No significant correlations were observed between the HepaAiQ score and tumor number (E). Sensitivity is higher in HCC patients with multiple tumor (F). BCLC, Barcelona Clinic Liver Cancer staging system.


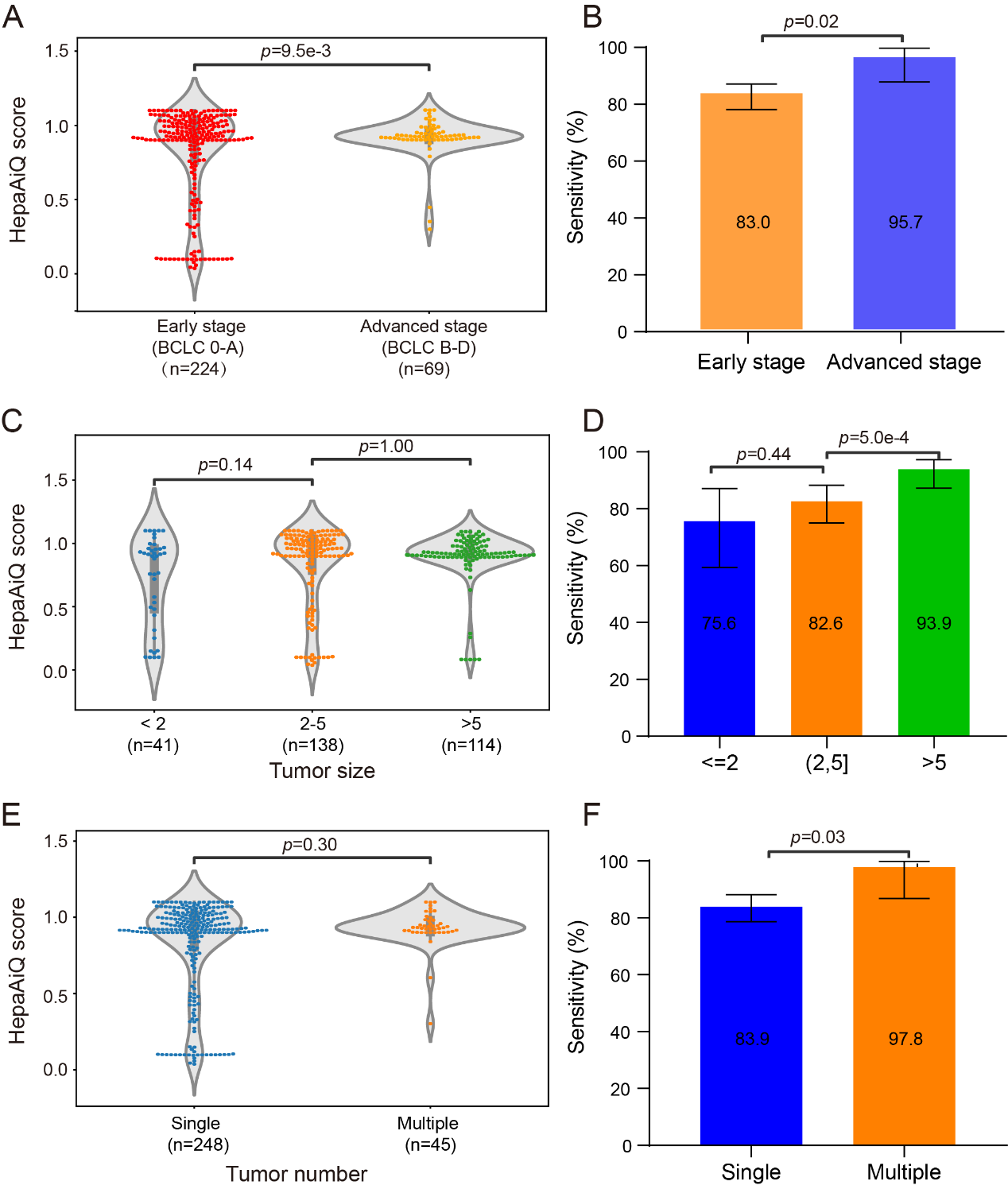


**Figure S3. Correlation analysis and sensitivity comparison for tumor stage, tumor size and tumor number of hepatocellular carcinoma patients in the validation cohort.** HepaAiQ score and sensitivity are higher in HCC patients with advanced stage (A, B). No significant correlations were observed between the HepaAiQ score and tumor size (C). Sensitivity is higher in HCC patients with tumor above 5cm (D). No significant correlations were observed between the HepaAiQ score and tumor number (E). Sensitivity is marginally higher in HCC patients with multiple tumor (F). BCLC, Barcelona Clinic Liver Cancer staging system.


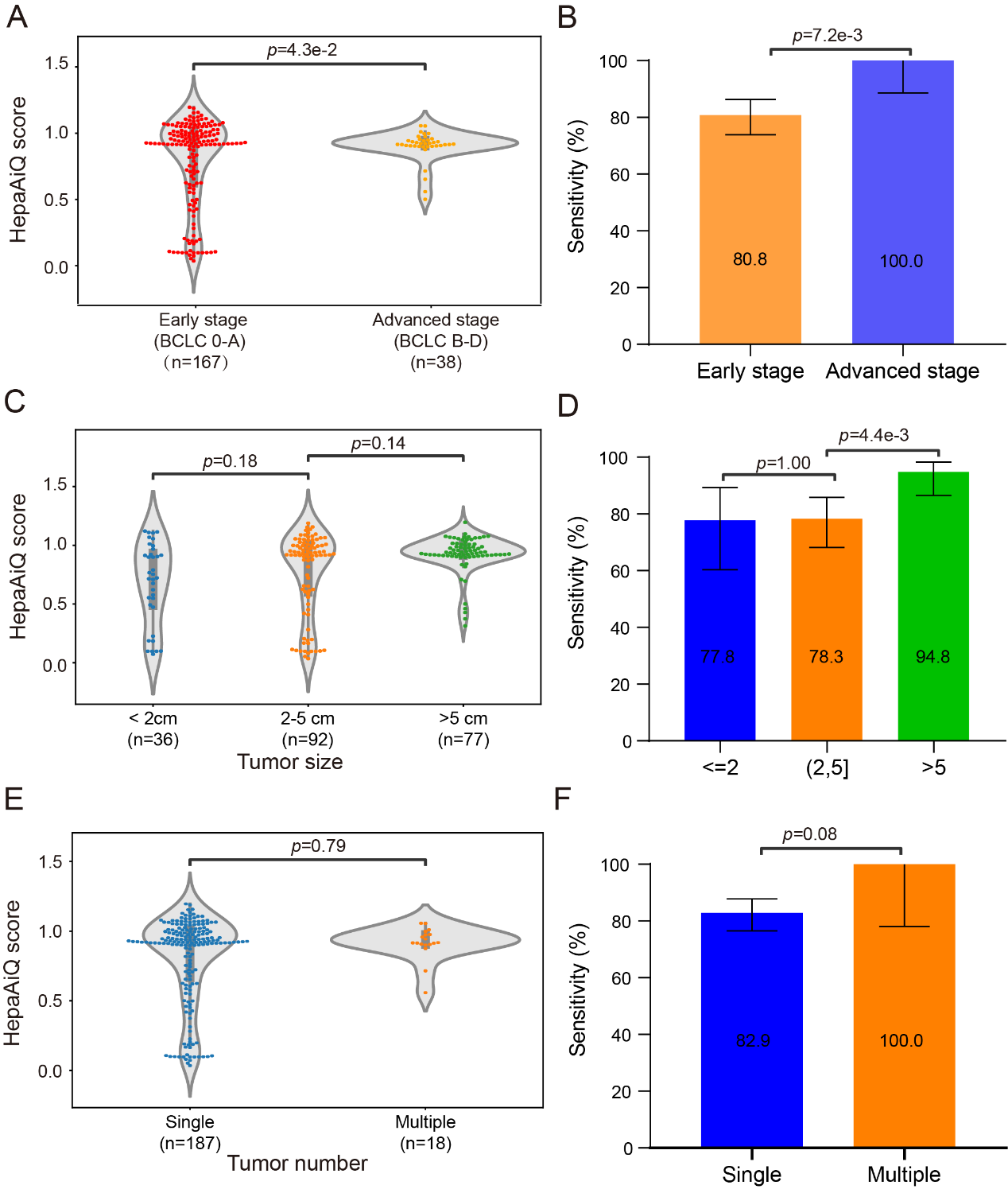


**Figure S4. Correlation analysis of HepaAiQ score with age and gender in the training cohort.** No significant correlations were observed between the HepaAiQ score and age (A) and gender (B). HepaAiQ score is higher in AFP-positive HCC patients than are the negative ones (C). No significant correlations were observed between the HepaAiQ score and DCP level (D). HCC: hepatocellular carcinoma.


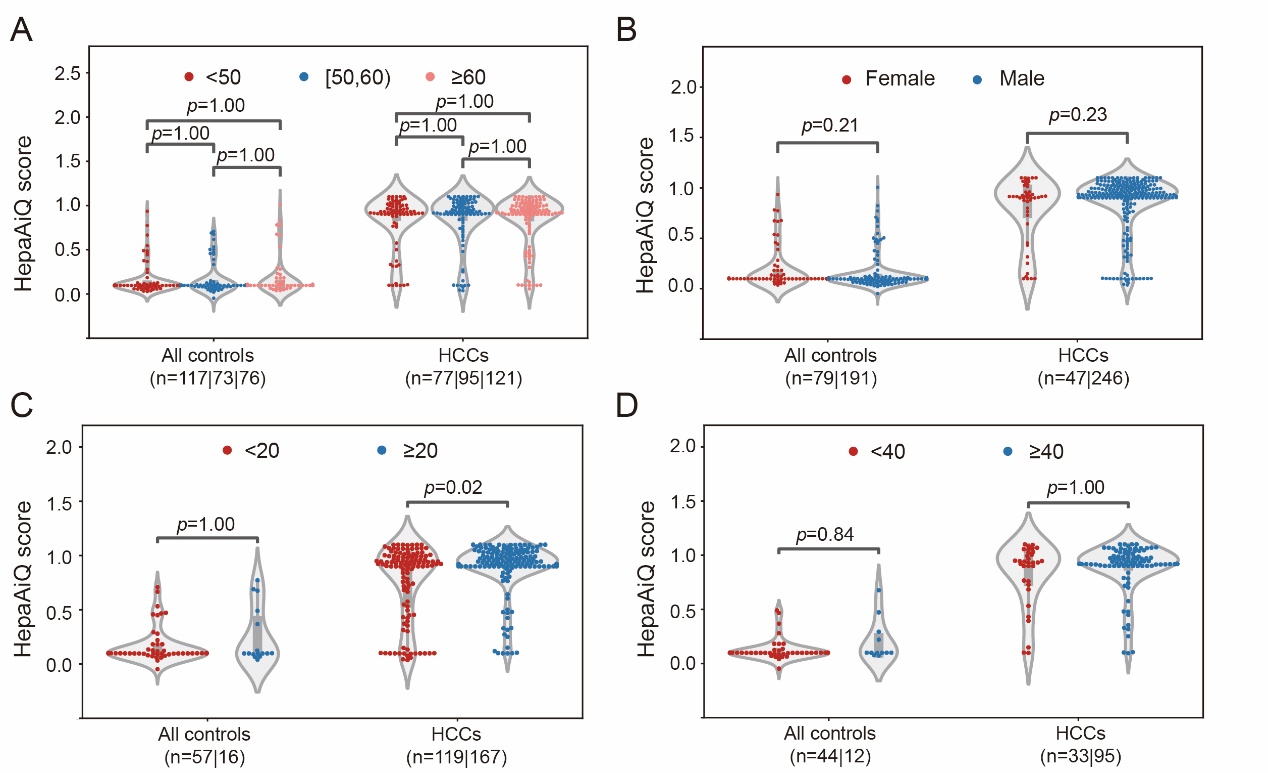


**Figure S5. Correlation analysis and sensitivity comparison for Child-Pugh grading of hepatocellular carcinoma patients in the training cohort.** No significant correlations were observed between the HepaAiQ score, or sensitivity and Child-Pugh grading (A, B).


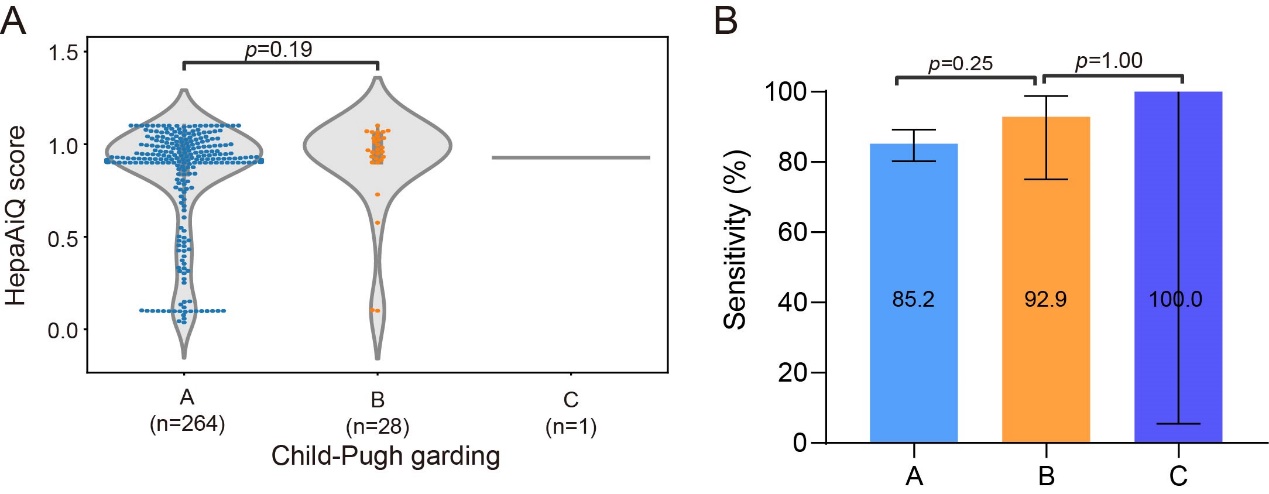


**Figure S6. Correlation analysis of HepaAiQ score with age and gender in the validation cohort.** No significant correlations were observed between the HepaAiQ score and age (A). No significant correlations were observed between the HepaAiQ score and gender in control groups. HepaAiQ score is higher in males than females in the HCC group (B). No significant correlations were observed between the HepaAiQ score and AFP level in control groups. HepaAiQ score is higher in AFP-positive HCC patients than are the negative ones (C). No significant correlations were observed between the HepaAiQ score and DCP level (D). AFP: alpha-fetoprotein; DCP: des-gamma-carboxy prothrombin. HCC: hepatocellular carcinoma.


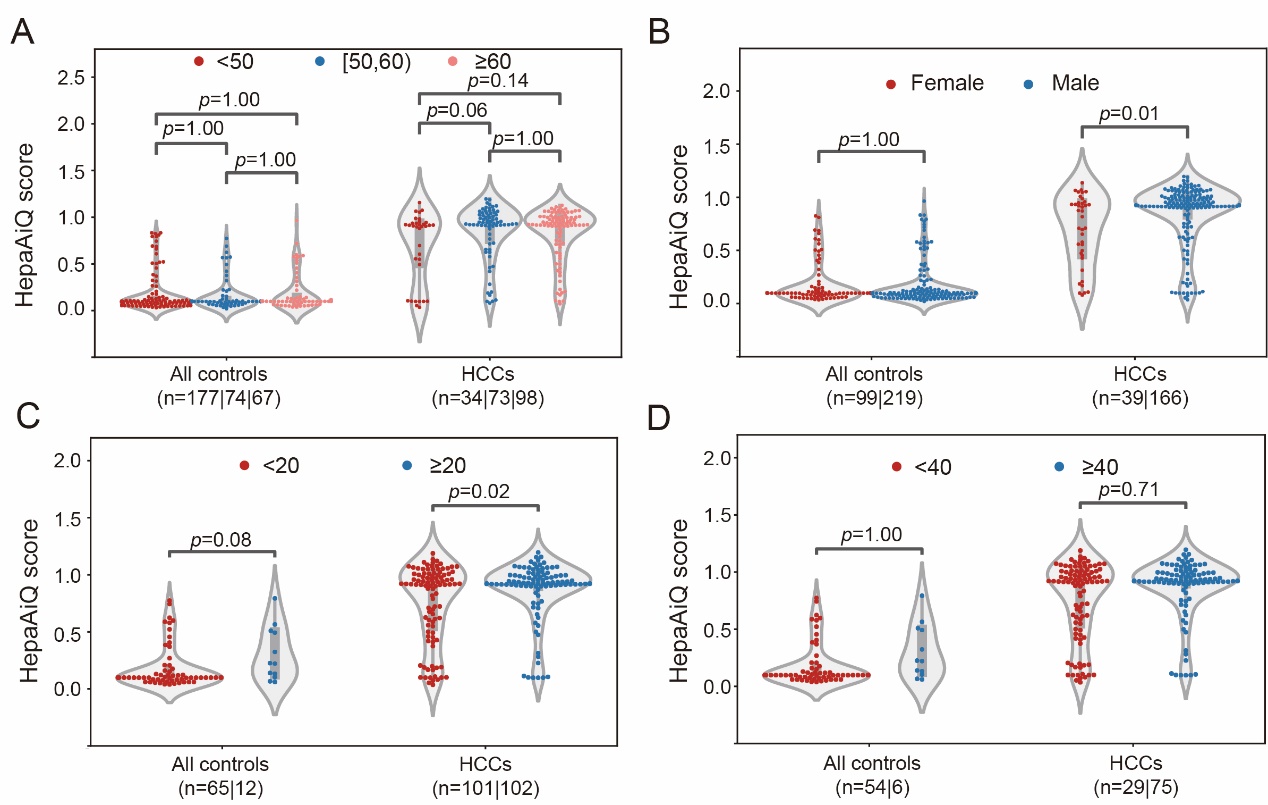


**Figure S7. Correlation analysis and sensitivity comparison for Child-Pugh grading of hepatocellular carcinoma patients in the validation cohort.** No significant correlations were observed between the HepaAiQ score, or sensitivity and Child-Pugh grading (A, B).


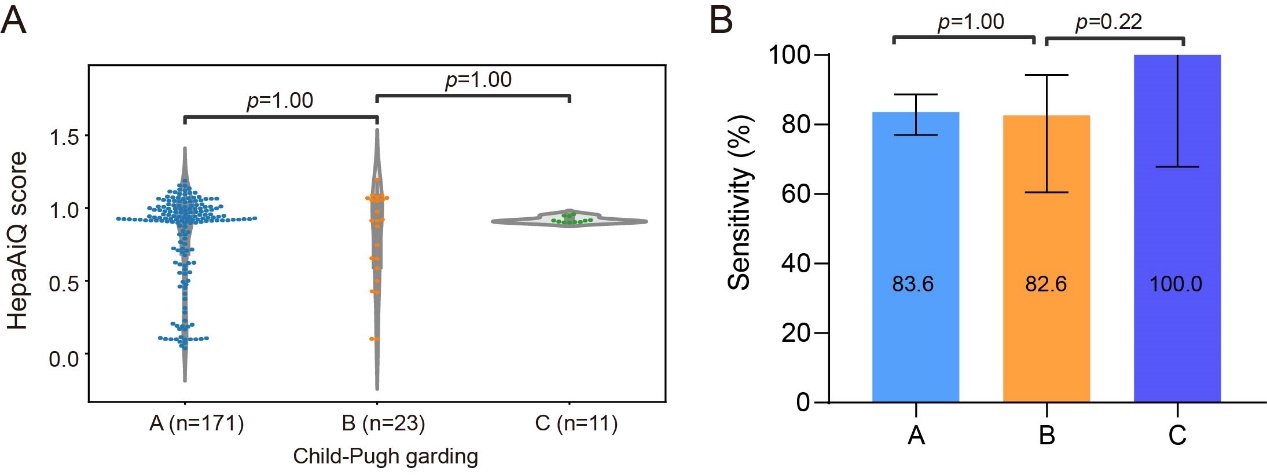


**Figure S8. HepaAiQ scores change in each recurrent patient and non-recurrent patient.** The dashed line in 0.471 indicates the cutoff of HepaAiQ scores. Pre-op, preoperation; Post-op, postoperation.


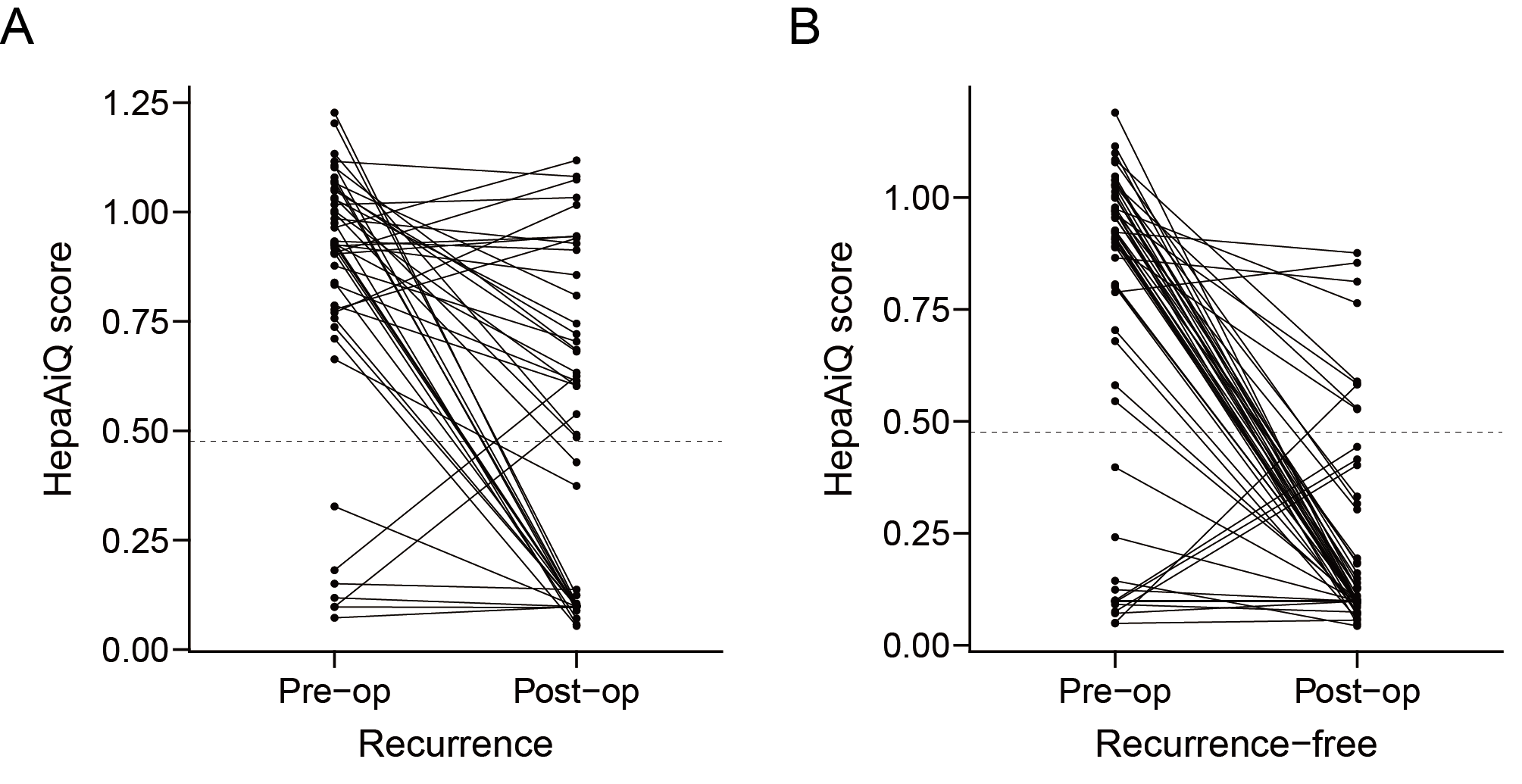


**Figure S9. Overall HepaAiQ score changes before and after surgery between patients with and without recurrence.** Recurrent patients exhibited a significantly higher postoperative ctDNA methylation level than non-recurrent patients. Pre-op, preoperation; Post-op, postoperation.


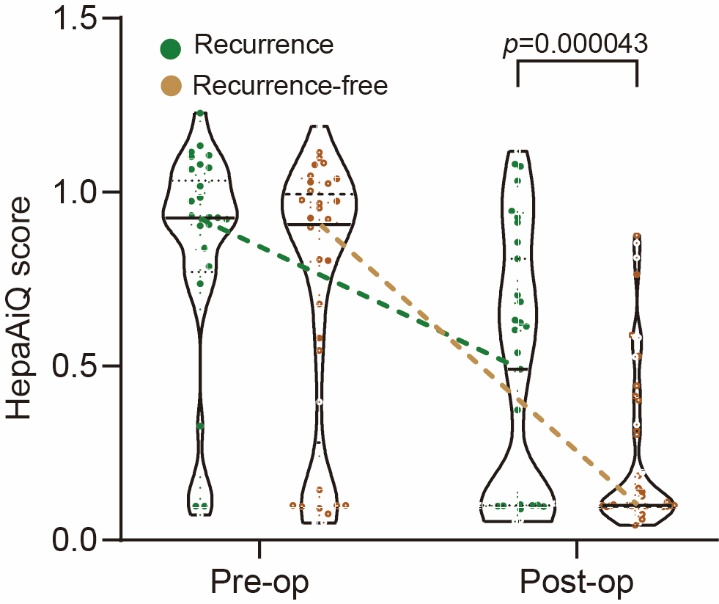


**Figure S10. Modeling the implementation of HepaAiQ in the HCC test.** A, We modeled the uncertainty in sensitivity and specificity for ultrasonography combined with AFP (US with AFP) as well as HepaAiQ screening in a high-risk theoretical population of 100,000 individuals. Predictive result of the number of detected HCC (B), positive predictive values (PPV) (C), and false negative rate (D), in a high-risk theoretical population. The Rank sum test was used to determine the p-value. US, ultrasonography; HCC, hepatocellular carcinoma; AFP, alpha-fetoprotein.


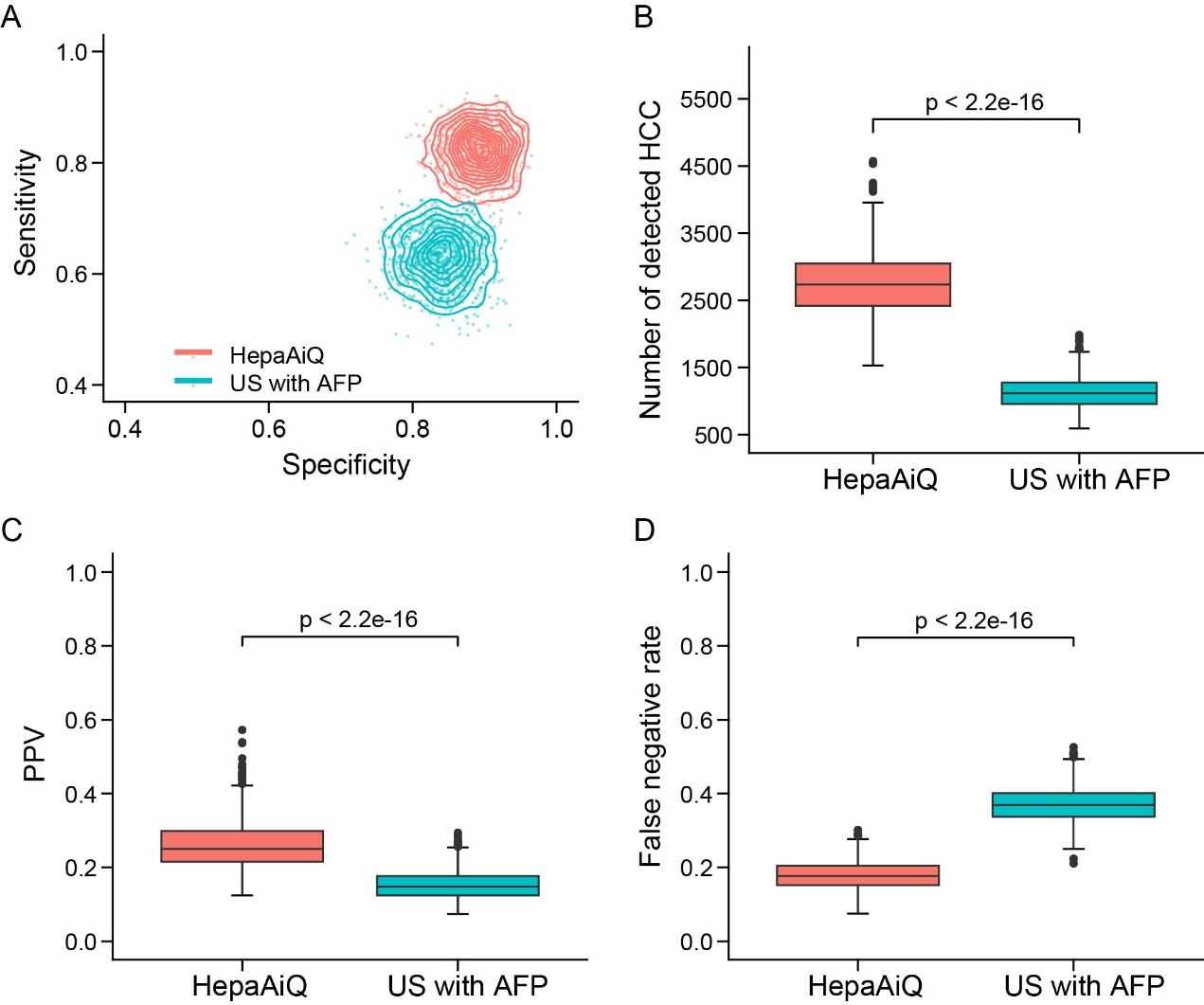


**Figure S11. Sensitivity of HepaAiQ model in HCC cases from different etiologies.** A, The sensitivities of the HepaAiQ model in HCC patients from different etiologies across training, validation, and test sets. B, Comparison of HepaAiQ sensitivities in HCC cases from different cohorts. No significant differences were observed between different etiologies. HCC, hepatocellular carcinoma.


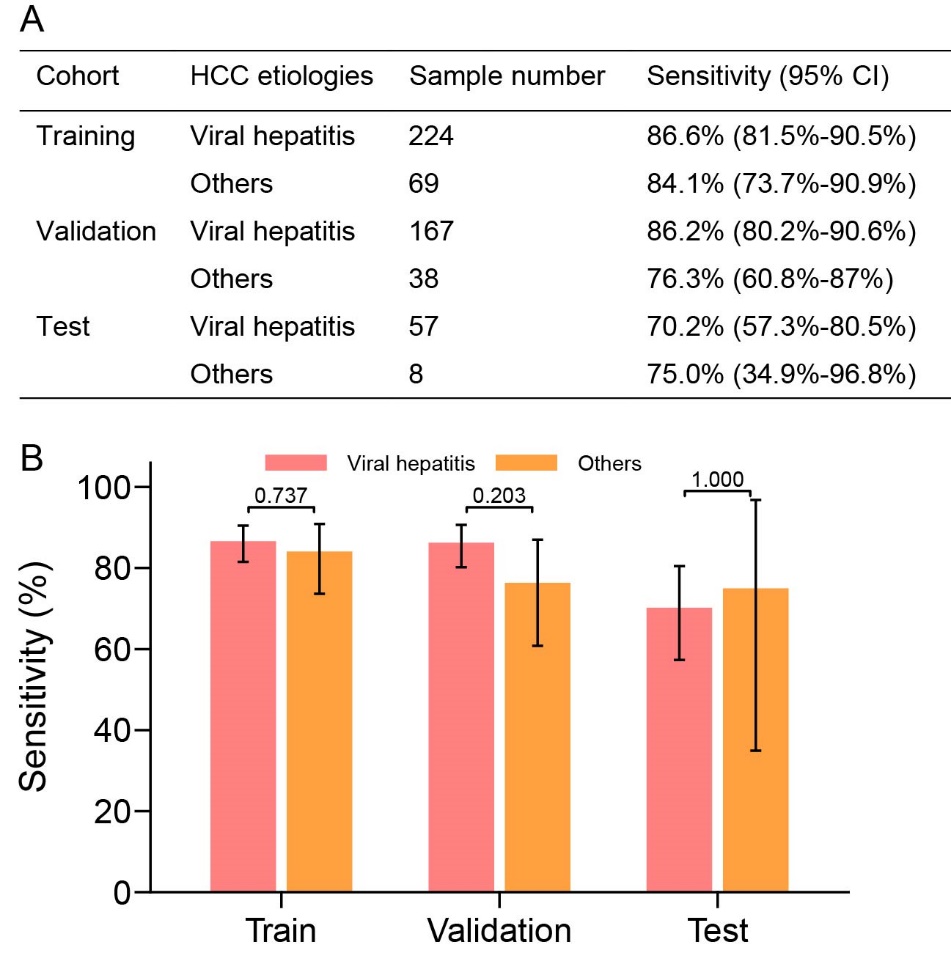


**Table S1. Top 20 best-performing markers selected from quantitative methylation-specific PCR.**

| Gene | WBC average Ct | WBC detection ratio | HCC tumor average Ct | HCC tumor detection ratio | Log10 fold change of HCC vs. WBC | *P value* |
| --- | --- | --- | --- | --- | --- | --- |
| BDH1 | 50.00 | 0% | 13.31 | 100.0% | 11.04 | 2.21E-24 |
| BDH1 region 2 | 50.00 | 0% | 14.10 | 100.0% | 10.81 | 3.49E-23 |
| GPAM | 50.00 | 0% | 15.73 | 100.0% | 10.32 | 9.47E-22 |
| NBPF10 | 50.00 | 0% | 18.48 | 100.0% | 9.49 | 1.10E-28 |
| CHFR | 50.00 | 0% | 18.96 | 100.0% | 9.35 | 2.76E-27 |
| B4GALNT1 | 50.00 | 0% | 19.26 | 100.0% | 9.25 | 1.73E-27 |
| BEND4 | 50.00 | 0% | 21.33 | 91.7% | 8.63 | 1.64E-11 |
| DAB2IP | 50.00 | 0% | 23.10 | 83.3% | 8.10 | 1.72E-09 |
| Septin9 region 2 | 50.00 | 0% | 23.64 | 91.7% | 7.94 | 2.37E-11 |
| BEST4 | 50.00 | 0% | 24.50 | 83.3% | 7.68 | 1.29E-08 |
| IRF4 | 48.80 | 0% | 24.33 | 91.7% | 7.37 | 2.20E-11 |
| GRASP | 50.00 | 0% | 25.65 | 83.3% | 7.33 | 6.00E-08 |
| IKZF1 | 50.00 | 0% | 29.98 | 75.0% | 6.03 | 1.19E-05 |
| Septin9 | 45.12 | 20% | 26.40 | 87.5% | 5.64 | 9.55E-09 |
| VASH2 | 50.00 | 0% | 32.60 | 58.3% | 5.24 | 8.14E-04 |
| SNX10 | 50.00 | 0% | 33.85 | 50.0% | 4.86 | 2.49E-03 |
| SPINT2 | 43.95 | 20% | 31.66 | 70.8% | 3.70 | 4.13E-03 |
| BCAT1 | 50.00 | 0% | 38.27 | 45.8% | 3.53 | 9.30E-03 |
| VAV3 | 50.00 | 0% | 38.51 | 41.7% | 3.46 | 1.05E-02 |
| TSPYL5 | 50.00 | 0% | 38.69 | 50.0% | 3.41 | 1.17E-03 |

WBC, white blood cell; HCC, hepatocellular carcinoma; PCR, polymerase chain reaction.

**Table S2. The performance of AFP and HepaAiQ.**

|  | **Total** | **Negative** | **Positive** | **Sensitivity (95% CI)** | **Specificity (95% CI)** | **PPV (95% CI)** | **NPV (95% CI)** |
| --- | --- | --- | --- | --- | --- | --- | --- |
| **HepaAiQ** |  |  |  |  |  | 94.6% (92.4%-96.7%) | 75.6% (70.7%-80.5%) |
| All HCCs | 489 | 72 | 417 | 85.3% (82.1%-88.4%) | **-** |  |  |
| BCLC 0-A (CNLC I) | 384 | 69 | 315 | 82.0% (78.2%-85.9%) | - |  |  |
| BCLC B-D (CNLC II-IV) | 105 | 3 | 102 | 97.1% (91.9%-99.0%) | - |  |  |
| All controls | 247 | 223 | 24 | **-** | 90.3% (86.6%-94%) |  |  |
| CHB/LC | 150 | 131 | 19 | - | 87.3% (82.0%-92.7%) |  |  |
| BHL | 97 | 92 | 5 | - | 94.8% (90.4%-99.2%) |  |  |
| **AFP** |  |  |  |  |  | 90.6% (87.2%-93.9%) | 49.9% (45.2%-54.6%) |
| All HCCs | 489 | 220 | 269 | 55.0% (50.6%-59.4%) | **-** |  |  |
| BCLC 0-A (CNLC I) | 384 | 189 | 195 | 50.8% (45.8%-55.8%) | - |  |  |
| BCLC B-D (CNLC II-IV) | 105 | 31 | 74 | 70.5% (61.8%-79.2%) | - |  |  |
| All controls | 247 | 219 | 28 | **-** | 88.7% (84.7%-92.6%) |  |  |
| CHB/LC | 150 | 122 | 28 | - | 81.3% (75.1%-87.6%) |  |  |
| BHL | 97 | 97 | 0 | - | 100.0% (96.2%-100.0%) |  |  |

HCC, hepatocellular carcinoma; BCLC, Barcelona Clinic Liver Cancer staging system; CNLC, the China Liver Cancer staging system; AFP, alpha-fetoprotein; CHB, chronic hepatitis B; LC, liver cirrhosis; BHL, benign hepatic lesion; CI, confidence interval.

**Table S3. The performance of HepaAiQ in AFP-Positive and AFP-negative samples.**

|  | **HepaAiQ** | | | | | | |
| --- | --- | --- | --- | --- | --- | --- | --- |
|  | **Total** | **Negative** | **Positive** | **Sensitivity (95% CI)** | **Specificity (95% CI)** | **PPV (95% CI)** | **NPV (95% CI)** |
| **AFP positive** |  |  |  |  |  | 96.9% (94.7%-99.0%) | 47.6% (32.5%-62.7%) |
| All HCCs | 269 | 22 | 247 | 91.8% (88.5%-95.1%) | **-** |  |  |
| BCLC 0-A (CNLC I) | 195 | 22 | 173 | 88.7% (84.3%-93.2%) | - |  |  |
| BCLC B-D (CNLC II-IV) | 74 | 0 | 74 | 100.0% (95.1%-100.0%) | - |  |  |
| All controls | 28 | 20 | 8 | **-** | 71.4% (52.9%-84.7%) |  |  |
| CHB/LC | 28 | 20 | 8 | - | 71.4% (52.9%-84.7%) |  |  |
| BHL | 0 | 0 | 0 | - | - |  |  |
| **AFP negative** |  |  |  |  |  | 91.4% (87.4%-95.4%) | 80.2% (75.3%-85.1%) |
| All HCCs | 220 | 50 | 170 | 77.3% (71.7%-82.8%) | **-** |  |  |
| BCLC 0-A (CNLC I) | 189 | 47 | 142 | 75.1% (69.0%-81.3%) | - |  |  |
| BCLC B-D (CNLC II-IV) | 31 | 3 | 28 | 90.3% (75.1%-96.7%) | - |  |  |
| All controls | 219 | 203 | 16 | **-** | 92.7% (89.2%-96.1%) |  |  |
| CHB/LC | 122 | 111 | 11 | - | 91.0% (85.9%-96.1%) |  |  |
| BHL | 97 | 92 | 5 | - | 94.8% (90.4%-99.2%) |  |  |

HCC, hepatocellular carcinoma; BCLC, Barcelona Clinic Liver Cancer staging system; CNLC, the China Liver Cancer staging system; AFP, alpha-fetoprotein; CHB, chronic hepatitis B; LC, liver cirrhosis; BHL, benign hepatic lesion; CI, confidence interval.

**Table S4. The performance of DCP and HepaAiQ.**

|  | **Total** | **Negative** | **Positive** | **Sensitivity (95% CI)** | **Specificity (95% CI)** | **PPV (95% CI)** | **NPV (95% CI)** |
| --- | --- | --- | --- | --- | --- | --- | --- |
| **HepaAiQ** |  |  |  |  |  | 91.6% (87.9%-95.2%) | 88% (83.6%-92.3%) |
| All HCCs | 232 | 26 | 206 | 88.8% (84.7%-92.9%) | **-** |  |  |
| BCLC 0-A (CNLC I) | 165 | 24 | 141 | 85.5% (80.1%-90.8%) | - |  |  |
| BCLC B-D (CNLC II-IV) | 67 | 2 | 65 | 97.0% (89.8%-99.2%) | - |  |  |
| All controls | 209 | 190 | 19 | **-** | 90.9% (87.0%-94.8%) |  |  |
| CHB/LC | 116 | 102 | 14 | - | 87.9% (82.0%-93.9%) |  |  |
| BHL | 93 | 88 | 5 | - | 94.6% (90.0%-99.2%) |  |  |
| **DCP** |  |  |  |  |  | 89.9% (85.6%-94.2%) | 75.1% (69.8%-80.4%) |
| All HCCs | 232 | 63 | 169 | 72.8% (67.1%-78.6%) | **-** |  |  |
| BCLC 0-A (CNLC I) | 165 | 56 | 109 | 66.1% (58.8%-73.3%) | - |  |  |
| BCLC B-D (CNLC II-IV) | 67 | 7 | 60 | 89.6% (82.2%-96.9%) | - |  |  |
| All controls | 209 | 190 | 19 | **-** | 90.9% (87.0%-94.8%) |  |  |
| CHB/LC | 116 | 98 | 18 | - | 84.5% (77.9%-91.1%) |  |  |
| BHL | 93 | 92 | 1 | - | 98.9% (94.2%-99.8%) |  |  |

HCC, hepatocellular carcinoma; BCLC, Barcelona Clinic Liver Cancer staging system; CNLC, the China Liver Cancer staging system; DCP, des-gamma-carboxyprothrombin; CHB, chronic hepatitis B; LC, liver cirrhosis; BHL, benign hepatic lesion; CI, confidence interval.

**Table S5. The performance of HepaAiQ in DCP-Positive and DCP-negative samples.**

|  | **HepaAiQ** | | | | | | |
| --- | --- | --- | --- | --- | --- | --- | --- |
|  | **Total** | **Negative** | **Positive** | **Sensitivity (95% CI)** | **Specificity (95% CI)** | **PPV (95% CI)** | **NPV (95% CI)** |
| **DCP positive** |  |  |  |  |  | 98.1% (94.6%-99.4%) | 55.2% (37.5%-71.6%) |
| All HCCs | 169 | 13 | 156 | 92.3% (88.3%-96.3%) | **-** |  |  |
| BCLC 0-A (CNLC I) | 109 | 12 | 97 | 89.0% (83.1%-94.9%) | - |  |  |
| BCLC B-D (CNLC II-IV) | 60 | 1 | 59 | 98.3% (91.1%-99.7%) | - |  |  |
| All controls | 19 | 16 | 3 | **-** | 84.2% (62.4%-94.5%) |  |  |
| CHB/LC | 18 | 15 | 3 | - | 83.3% (60.8%-94.2%) |  |  |
| BHL | 1 | 1 | 0 | - | 100.0% (20.7%-100.0%) |  |  |
| **DCP negative** |  |  |  |  |  | 75.8% (65.4%-86.1%) | 93% (89.4%-96.7%) |
| All HCCs | 63 | 13 | 50 | 79.4% (69.4%-89.4%) | **-** |  |  |
| BCLC 0-A (CNLC I) | 56 | 12 | 44 | 78.6% (67.8%-89.3%) | - |  |  |
| BCLC B-D (CNLC II-IV) | 7 | 1 | 6 | 85.7% (48.7%-97.4%) | - |  |  |
| All controls | 190 | 174 | 16 | **-** | 91.6% (87.6%-95.5%) |  |  |
| CHB/LC | 98 | 87 | 11 | - | 94.6% (89.9%-99.2%) |  |  |
| BHL | 92 | 87 | 5 | - | 91.6% (87.6%-95.5%) |  |  |

HCC, hepatocellular carcinoma; BCLC, Barcelona Clinic Liver Cancer staging system; CNLC, the China Liver Cancer staging system; DCP, des-gamma-carboxyprothrombin; CHB, chronic hepatitis B; LC, liver cirrhosis; BHL, benign hepatic lesion; CI, confidence interval.

**Table S6. Patient demographics and clinical information in the independent test cohort.**

| Variable | HCC (n=65) | Controls (n=124) |
| --- | --- | --- |
| Age median (Min, Max) | 57.0 (33.0, 80.0) | 48.0 (30.0, 73.0) |
| Age (years) |  |  |
| ≤50 | 22 (33.8%) | 71 (57.3%) |
| >50 | 43 (66.2%) | 53 (42.7%) |
| Gender |  |  |
| Male | 49 (75.4%) | 78 (62.9%) |
| Female | 16 (24.6%) | 46 (37.1%) |
| Liver disease |  |  |
| CHB/LC | 65 | 124 |
| BCLC stage (CNLC stage) |  |  |
| 0-A (I) | 65 | - |

HCC, hepatocellular carcinoma; BCLC, Barcelona Clinic Liver Cancer staging system; CNLC, the China Liver Cancer staging system; CHB, chronic hepatitis B; LC, liver cirrhosis.

**Table S7. The performance of AFP and HepaAiQ in the independent test cohort.**

|  | **Total** | **Negative** | **Positive** | **Sensitivity (95% CI)** | **Specificity (95% CI)** |
| --- | --- | --- | --- | --- | --- |
| **HepaAiQ** |  |  |  |  |  |
| All HCCs | 65 | 19 | 46 | 70.8% (59.7%-81.8%) | **-** |
| BCLC 0-A (CNLC I) | 65 | 19 | 46 | 70.8% (59.7%-81.8%) | - |
| All controls | 110 | 100 | 10 | - | 90.9% (85.5%-96.3%) |
| CHB/LC | 110 | 100 | 10 | - | 90.9% (85.5%-96.3%) |
| **AFP** |  |  |  |  |  |
| All HCCs | 65 | 27 | 38 | 58.5% (46.5%-70.4%) | - |
| BCLC 0-A (CNLC I) | 65 | 27 | 38 | 58.5% (46.5%-70.4%) | - |
| All controls | 110 | 102 | 8 | - | 92.7% (87.9%-97.6%) |
| CHB/LC | 110 | 102 | 8 | - | 92.7% (87.9%-97.6%) |

AFP, alpha-fetoprotein; HCC, hepatocellular carcinoma; BCLC, Barcelona Clinic Liver Cancer staging system; CNLC, the China Liver Cancer staging system; CHB, chronic hepatitis B; LC, liver cirrhosis; CI, confidence interval.

**Table S8. Clinical characteristics of prognosis assessment cohort.**

| Variable | Recurrence (n=47) | Recurrence-free  (n=56) | *P* value |
| --- | --- | --- | --- |
| Age (years) |  |  | 1.000 |
| ≤50 | 14 (29.8%) | 17 (30.4%) |  |
| >50 | 33 (70.2%) | 39 (69.6%) |  |
| Gender |  |  | 0.189 |
| Male | 43 (91.5%) | 45 (80.4%) |  |
| Female | 4 (8.5%) | 11 (19.6%) |  |
| Child-Pugh class |  |  | 0.086 |
| A | 43 (91.5%) | 56 (100%) |  |
| B-C | 4 (8.5%) | 0 (0%) |  |
| BCLC stage (CNLC stage) |  |  | 0.081 |
| 0-A (I) | 36 (76.6%) | 51 (91.1%) |  |
| B-D (II-IV) | 11 (23.4%) | 5 (8.9%) |  |
| Tumor size |  |  | 0.444 |
| ≤5 | 32 (68.1%) | 43 (76.8%) |  |
| >5 | 15 (31.9%) | 13 (23.2%) |  |
| Tumor number |  |  | 0.070 |
| Single | 41 (87.2%) | 55 (98.2%) |  |
| Multiple | 6 (12.8%) | 1 (1.8%) |  |
| Preop AFP (ng/ml) |  |  | 0.680 |
| ≤20 | 19 (40.4%) | 26 (46.4%) |  |
| >20 | 28 (59.6%) | 30 (53.6%) |  |
| Preop DCP (μg/L) |  |  | 0.187 |
| ≤40 | 7 (14.9%) | 15 (26.8%) |  |
| >40 | 39 (83.0%) | 38 (67.9%) |  |
| No test | 1 (2.1%) | 3 (5.4%) |  |
| Preop HepaAiQ |  |  | 0.220 |
| Negative | 7 (14.9%) | 15 (26.8%) |  |
| Positive | 40 (85.1%) | 41 (73.2%) |  |
| Postop HepaAiQ |  |  | **<0.001** |
| Negative | 22 (46.8%) | 47 (83.9%) |  |
| Positive | 25 (53.2%) | 9 (16.1%) |  |

AFP, alpha-fetoprotein; DCP, des-gamma-carboxy prothrombin. CNLC, the China Liver Cancer staging system; BCLC, the Barcelona Clinic Liver Cancer staging system; Preop, preoperative; Postop, postoperative.

**Table S9. Univariate and multivariate Cox regression analysis for possible variables associated with recurrence in prognosis assessment cohort.**

|  | Univariate | |  | Multivariate | |
| --- | --- | --- | --- | --- | --- |
| Variable | HR (95% CI) | *P* value |  | HR (95% CI) | *P* value |
| Age (>50/≤50) | 1.04 (0.55-1.98) | 0.898 |  | - | - |
| Gender (Male/Female) | 2.02 (0.72-5.63) | 0.179 |  | - | - |
| Tumor size (>5/≤5) | 1.63 (0.88-3.03) | 0.119 |  | - | - |
| Tumor number (Multiple/Single) | 3.19 (1.35-7.57) | **0.008** |  | 2.63 (1.10-6.28) | 0.030 |
| Preop AFP (ng/ml)  (>20/≤20) | 1.17 (0.65-2.11) | 0.608 |  | - | - |
| Preop DCP (ug/L)  (>40/≤40) | 1.89 (0.85-4.24) | 0.120 |  | - | - |
| Preop HepaAiQ (Positive/Negative) | 1.79 (0.80-4.01) | 0.155 |  | - | - |
| Postop HepaAiQ (Positive/Negative) | 3.30 (1.84-5.90) | **<0.001** |  | 3.13 (1.74-5.64) | <0.001 |

AFP, alpha-fetoprotein; DCP, des-gamma-carboxy prothrombin; Preop, preoperative; Postop, postoperative; HR, hazard ratio; CI, confidence interval.

Reference:

1. Mooney, C.Z., *Monte carlo simulation*. 1997: Sage.

2. Tzartzeva, K., et al., *Surveillance Imaging and Alpha Fetoprotein for Early Detection of Hepatocellular Carcinoma in Patients With Cirrhosis: A Meta-analysis.* Gastroenterology, 2018. **154**(6): p. 1706-1718.e1.

3. Xiao, J., et al., *Global liver disease burdens and research trends: Analysis from a Chinese perspective.* J Hepatol, 2019. **71**(1): p. 212-221.

4. Xie, D.Y., et al., *A review of 2022 Chinese clinical guidelines on the management of hepatocellular carcinoma: updates and insights.* Hepatobiliary Surg Nutr, 2023. **12**(2): p. 216-228.

5. Shi, J.F., et al., *[Access to liver cancer screening and surveillance in populations in China: an exploratory analysis].* Zhonghua Liu Xing Bing Xue Za Zhi, 2022. **43**(6): p. 906-914.

6. Adler, A., et al., *Improving compliance to colorectal cancer screening using blood and stool based tests in patients refusing screening colonoscopy in Germany.* BMC Gastroenterol, 2014. **14**: p. 183.

7. Bokhorst, L.P., et al., *Compliance Rates with the Prostate Cancer Research International Active Surveillance (PRIAS) Protocol and Disease Reclassification in Noncompliers.* Eur Urol, 2015. **68**(5): p. 814-21.
